# “Surviving Discrimination by Pulling Together”: LGBTQI Cancer Patient and Carer Experiences of Minority Stress and Social Support

**DOI:** 10.3389/fonc.2022.918016

**Published:** 2022-06-24

**Authors:** Rosalie Power, Jane M. Ussher, Janette Perz, Kimberley Allison, Alexandra J. Hawkey

**Affiliations:** Translational Health Research Institute, School of Medicine, Western Sydney University, Sydney, NSW, Australia

**Keywords:** cancer, LGBTQI, minority stress, social support, discrimination, intersex, transgender (binary and non-binary), adolescents and young adults (AYA)

## Abstract

**Background:**

Lesbian, gay, bisexual, transgender, queer and/or intersex (LGBTQI) people with cancer and their carers report poorer psychological outcomes than the general non-LGBTQI cancer population. There is growing acknowledgement that these health inequities can be explained by minority stress, which can be buffered by social support.

**Study Aim:**

To examine subjective experiences of minority stress and social support for LGBTQI people with cancer and their carers, drawing on qualitative findings from the Out with Cancer study.

**Method:**

An online survey including open ended items was completed by 430 LGBTQI cancer patients and 132 partners and other carers, representing a range of tumor types, sexual and gender identities, age and intersex status. A sub-sample of 104 patients and 31 carers completed an interview, with a follow-up photovoice activity and second interview completed by 45 patients and 10 carers. Data was thematically analysed using an intersectional theoretical framework.

**Results:**

Historical and present-day experiences of discrimination, violence, family rejection and exclusion created a legacy of distress and fear. This impacted on trust of healthcare professionals and contributed to distress and unmet needs in cancer survivorship and care. Social support, often provided by partners and other chosen family, including intimate partners and other LGBTQI people, buffered the negative impacts of minority stress, helping LGBTQI patients deal with cancer. However, some participants lacked support due to not having a partner, rejection from family of origin and lack of support within LGBTQI communities, increasing vulnerability to poor psychological wellbeing. Despite the chronic, cumulative impacts of minority stress, LGBTQI patients and carers were not passive recipients of discriminatory and exclusion in cancer care, demonstrating agency and resistance through collective action and advocacy.

**Conclusion:**

LGBTQI people have unique socio-political histories and present-day psycho-social experiences that contribute to distress during cancer. Social support serves to buffer and ameliorate this distress. There is a need for cancer healthcare professionals and support services to be aware of and responsive to these potential vulnerabilities, including the intersectional differences in experiences of minority stress and social support. There is also a need for recognition and facilitation of social support among LGBTQI people with cancer and their carers.

## 1 Introduction

The experience of cancer diagnosis, treatment and survivorship can be very stressful for people with cancer and their carers, leading to depression and anxiety (1). There is growing evidence that lesbian, gay, bisexual, transgender queer and/or intersex (LGBTQI) people who have experienced cancer, and their carers, report poorer mental health outcomes than the general non-LGBTQI cancer population. This includes higher levels of cancer-related distress (2, 3), and higher depression and anxiety (4, 5). To date, most LGBTQI cancer research has focused on adult cisgender individuals with breast or prostate cancer (6). Recent systematic literature reviews highlight the need to understand the complexity of LGBTQI experience of cancer across cancer streams, age, and sexual identity subgroups, including people who are trans (binary and non-binary) and intersex (4, 7–9). An intersectional theoretical framework has been recommended (10) to analyze how the complex spheres of identity intersect (11) to affect health outcomes among LGBTQI people with cancer (4, 10, 12).

There is also a need to examine the perspectives and experiences of informal carers, who are often invisible within LGBTQI cancer research and care (13). Caring for a partner, family member or close friend with cancer can have significant negative consequences for health and wellbeing (14, 15). LGBTQI caregivers report higher caregiving burden and unique support needs compared to non-LGBTQI caregivers, including experiences of minority stress and lack of inclusion in cancer care (16–18). In comparison to the general cancer population, LGBTQI people with cancer are more likely to be unpartnered, and to receive support from ‘chosen family’, including friends and other LGBTQI people (2, 19). There is evidence that LGBTQI chosen family caregivers experience the same levels of stress as partner caregivers, yet they often lack access to social support, increasing their vulnerability to poor psychological wellbeing (20, 21).

Health inequities reported by LGBTQI people with cancer and their carers can be explained by minority stress theory (3, 22, 23). Minority stress is the experience of chronic stress associated with living with a marginalized LGBTQI identity. It is manifested by experience and anticipation of stigma, exclusion, discrimination, and violence (described as distal stressors). The internalization of anti-LGBTQI sentiments contributes to negative self-views, identity concealment and expectations of rejection, hostility and potential future victimization (described as proximal stressors) (22–24). Supporting this theory, distress reported by LGBTQI cancer patients is significantly associated with experiences of discrimination in life and cancer care (3, 25, 26), accompanied by identity concealment and expectations of future hostility (27). LGBTQI people report lower satisfaction with cancer care (28) and greater unmet care needs (29) in comparison with the general cancer population. This includes a lack of adequate information and support (6), and reluctance to ‘come out’ in healthcare settings due to fear of discrimination (30).

Previous research has reported that LGBTQI people experience high rates of discrimination in their everyday lives, including physical and sexual violence (31, 32), and hostile social environments that compromise wellbeing (33, 34). Throughout history, people now referred to as LGBTQI have been viewed as immoral, enabling their relationships and bodies to be subject to state-sanctioned violence and systemic injustice (35, 36) including discrimination in healthcare during the HIV/AIDS epidemic of the 1980s (37). Until 1973, homosexuality was classified as a mental illness by the American Psychiatric Association (38), and until 1991 consensual homosexual acts were criminalized in parts of Australia (39), the primary site for the present study. There is continued pathologization of trans and intersex people within mainstream health systems (40, 41). LGBTQI people’s behaviors remain criminalized, sometimes punishable by death, in many parts of the world (42). LGBTQI people are also subject to hostile public and political discourses that legitimize discrimination and attempt to roll back LGBTQI human rights (43–46), with negative implications for mental health and feelings of safety (47, 48).

Social support can buffer the negative impacts of distal and proximal minority stressors (3, 49) and is associated with improved quality of life (15, 50) and reduced distress (51), depression and anxiety (5) in cancer survivorship and caregiving. However, LGBTQI people may lack social support, due to having no intimate partner, experiences of family rejection, or the impact of stigma and social exclusion where they live (24, 52). Low social support has been found to be a unique predictor of distress in LGBTQI cancer patients (3, 53). However, the subjective meanings and experiences of minority stress and the ways in which social support may ameliorate or buffer distress for LGBTQI cancer patients and their carers within intersecting identities remains unexplored (8).

The present analysis aimed to address this gap in the research literature by examining subjective experiences of minority stress and social support among LGBTQI people with cancer and their carers, drawing on the qualitative findings from the mixed method Out with Cancer study (27, 53–55). This complements quantitative analysis from this study, which found higher rates of distress among LGBTQI people with cancer compared with general cancer populations, associated with minority stress and lack of social support (53). High rates of minority stress were reported by both LGBTQI cancer patients and their carers, including discrimination in life and in cancer care, and concealment of LGBTQI identity (53, 55).

This paper enables further interpretation of these findings, through in-depth qualitative examination of the nature and perceived impact of minority stress and social support for LGBTQI cancer survivors and their carers.

## 2 Methods

### 2.1 Study Design and Theoretical Framework

This study was part of the broader mixed methods Out with Cancer project (27, 53–55). The project examined LGBTQI experiences of cancer from the perspectives of LGBTQI patients, their caregivers, and healthcare professionals (HCPs), in order to inform LGBTQI inclusive cancer care. This paper presents the qualitative analysis of open-ended survey responses, interviews and a photovoice activity, related to LGBTQI cancer patient and carer experiences of minority stress and social support.

The project uses an intersectional theoretical framework, which acknowledges that individuals inhabit multiple interconnected social identity categories, such as gender, sexuality, cultural background and age (56). These identity categories are embedded in systems of social stratification, associated with power inequalities (57–59), and influence social practices and health and wellbeing (60). An intersectional perspective recognizes that identity cannot be reduced to the summary of social groups to which a person belongs; instead, attention is paid to how social identities interact to produce a meaningful whole in a way that cannot be explained by looking at one social identity alone (58), and influence social practices and health and wellbeing (60).

Integrated knowledge translation (iKT), a dynamic collaborative process between researchers and knowledge users to achieve actionable research outcomes, guided the study design, data collection, analysis and dissemination. Following principles of iKT, a steering committee comprising LGBTQI people with cancer, cancer HCPs, and representatives from LGBTQI health and cancer support organizations, were actively involved through co-design in all stages of the study. Discussion between the researchers and the steering group facilitated reflexivity (61), critical evaluation of the ways in which our positions as LGBTQI people, clinicians, researchers, and/or cancer survivors influenced the research process and outcome. The study received ethics approval from Western Sydney University Human Research Ethics Committee (ref. no. H12664, with secondary approval from the ACON (formerly the AIDS Council of New South Wales) (ref. no. 2019/09).

### 2.2 Recruitment

Participants were eligible for this study if they: (a) had been diagnosed with cancer, had undergone a medical intervention related to cancer risk or had cared for someone with cancer; (b) they or the person they cared for identified as LGBTQI, and (c) were at least 15 years old. Participants were recruited through cancer and LGBTQI community organizations, including the study partner organizations, social media (Facebook, Twitter, Instagram), cancer research databases (Register 4, ANZUP), cancer support groups and LGBTQI community events. Snowball sampling was also used, asking participants to pass the study information to someone they knew who fitted the study criteria. The study was open internationally, although recruitment focused on Australia and other English-speaking countries such as the USA, UK, New Zealand, and Canada. Recruitment strategies engaged LGBTQI patients and their carers in a range of sexual and gender identities, ages and tumor types. Individual strategies were used for each LGBTQI sub-group and to engage participants from intersecting minority backgrounds, including Indigenous Australians, people from migrant backgrounds, and adolescents and young adults (AYAs). Data were collected between September 2019 and September 2021.

### 2.3 Participants and Procedure

Participants took part in a three-stage study: A total of 430 LGBTQI people (patients) who currently or previously had cancer (82.8%), or a medical intervention related to cancer risk (17.2%), and 132 partners and other carers (hereafter, carers), aged 15 years or older, completed an online survey. A subset of survey participants, 104 patients and 31 carers, completed a 60-minute interview to investigate their experiences in greater depth. Forty-five patients and ten carers completed an additional photovoice activity.

Demographic characteristics of patients and carers are reported in **Table 1**. Most patients and carers were cis women, Caucasian, older adults, living in Australia and identified as lesbian, gay, or homosexual. Greater diversity was evident in participants’ regionality and cancer types. A minority of participants identified as trans (binary and non-binary; hereafter trans), bisexual or pansexual, queer, reported an intersex variation, or were AYA. A minority identified as Indigenous Australian, or Māori, Asian, or from a mixed ethnic background. Most carers were partners of LGBTQI people with cancer (63.6%).

**Table 1:** Demographic characteristics of LGBTQI patients and carers.

	Patients (*n*=430)	Carers (*n*=132)
Age at time of study (mean years, standard deviation; range)	*M=*52.5, *sd=*15.7; range 16-92	*M=*50.2 *sd=*17.0; range 15-76
	*n* (%)	*n* (%)
Country		
* Australia*	311 (72.3%)	93 (70.5%)
* United States of America *	62 (14.4%)	14 (10.6%)
* United Kingdom*	29 (6.7%)	9 (6.8%)
* New Zealand*	8 (1.9%)	6 (4.5%)
* Canada*	7 (1.6%)	4 (3.0%)
* Other country*	13 (3.0%)	6 (3.6%)
Race/ethnicity		
* Caucasian*	362 (85.2%)	109 (82.6%)
* Asian *	11 (2.6%)	5 (3.8%)
* Australian Aboriginal, Torres Strait Islander or Māori*	9 (2.1%)	4 (3.0%)
* Mixed background*	19 (4.5%)	6 (4.5%)
* Other/unclear background*	24 (5.6%)	8 (6.1%)
Location		
* Urban*	234 (54.5%)	69 (52.3%)
* Regional*	145 (33.8%)	48 (36.4%)
* Rural or remote*	50 (11.7%)	15 (11.4%)
Gender		
* Cis female*	216 (50.2%)	83 (62.9%)
* Cis male*	145 (33.7%)	26 (19.7%)
* Trans (binary and non-binary)^1^*	63 (14.7%)	23 (17.4%)
* Different gender identity*	6 (1.4%)	–
Sexuality		
* Lesbian, gay or homosexual*	317 (73.7%)	95 (72.0%)
* Bisexual or pansexual*	47 (10.9%)	17 (12.9%);
* Queer*	45 (10.5%)	12 (9.1%)
* Straight or heterosexual*	10 (2.3%)	5 (3.8%)
* Different or multiple identities*	11 (2.6%)	3 (2.3%)
Intersex variation		
* Yes*	31 (7.2%)	5 (3.8%)
* No*	388 (90.2%)	127 (96.2%)
* Prefer not to answer *	11 (2.6%)	0
Cancer Type^2^		
* Brain*	11 (3.0%)	9 (7.0%)
* Breast*	90 (24.3%)	37 (28.7%)
* Cervical*	11 (3.0%)	4 (3.1%)
* Colorectal*	17 (4.6%)	8 (6.2%)
* Head/neck*	14 (3.8%)	10 (7.8%)
* Leukaemia*	17 (4.6%)	5 (3.9%)
* Lymphoma*	24 (6.5%)	6 (4.7%)
* Ovarian*	17 (4.6%)	13 (10.1%)
* Prostate*	59 (15.9%)	8 (6.2%)
* Skin*	25 (6.8%)	3 (2.3%)
* Uterine*	23 (6.2%)	4 (3.1%)
* Other*	58 (15.7%)	19 (14.7%)
* Not sure or unknown*	4 (1.1%)	3 (2.3%)
Medical intervention for cancer risk	74 (17.2%)	27 (22.9%)
Carer relationship to patient		
* Partner/ex-partner*	–	84 (63.6%)
* Family*	–	31 (23.5%)
* Friend*	–	12 (9.1%)
* Different relationship*	–	3 (2.3%)
* Multiple care relationships*	–	2 (1.5%)

^1^Patients: 34 (7.9%) non-binary, 13 (3.0%) trans female, 8 (1.9%) trans male, 8 (1.9%) different TGD identity; Carers: 16 (12.1%) non-binary, 5 (3.8%) trans female, 2 (1.5%) trans male.

^2^Cancer type for carers is of patient cared for.

### 2.4 Materials

#### 2.4.1 Survey

The survey comprised a series of closed and open-ended measures. Full details of the patient (53) and carer (55) surveys are described in detail elsewhere. This paper focuses on responses to open-ended questions on minority stress and social support. Following quantitative measures about minority stress (discrimination in life and in cancer care, internalized prejudice and identity concealment) and social support (access to support from others), participants were asked “is there anything you would like to tell us about this issue?”.

#### 2.4.2 Semi-Structured Interview

Semi-structured, one-to-one interviews, using a conversational style, were undertaken by a number of researchers to explore subjective experiences in-depth. Interviews were conducted over the telephone or using video-conferencing software with LGBTQI patients and their carers. These were audio-recorded. Participants were asked about their experiences of cancer, including interactions with HCPs, decision-making pertaining to disclosure of their LGBTQI status and the consequences of this for their cancer care; the impact of cancer on their lives, including on their identities and relationships; experiences of finding support networks and information as an LGBTQI cancer patient.

#### 2.4.3 Photovoice Activity

Interview participants were invited to engage in a photovoice activity. Photovoice involves participants taking photographs that visualise elements within an individual’s life pertinent to a particular phenomenon (62). Situated within an action-research model, photovoice methods facilitate involvement and empowerment of research participants and have been described as an innovative way of working with marginalised people, including LGBTQI communities (63, 64). The method implicitly challenges traditional structures of power and traditional modes of knowledge production (65). Participants were invited to submit three to five photographs that represented their experiences with cancer, which were then discussed in a second interview. Written and visual instructions were provided to participants to aid in the photovoicep process. Participants used their devices (smartphones, digital cameras) to take photographs and electronically submit them to the research team. The photographs were used as the basis for subsequent discussions to understand more about participants’ experiences of cancer and often eliciting in-depth descriptions of specific events. Participants were asked questions such as “Could you please talk me through these photos and explain what they mean to you?” and “How does this photo capture your cancer experience?”. All participants provided informed consent for their photographs to be used in analysis and in publications.

### 2.5 Data Analysis

Thematic analysis was used to analyse the open-ended survey, interview and photovoice data, as an appropriate method to capture richness across multiple data types (66). All interviews were professionally transcribed verbatim, and integrity checked for any errors. The transcripts were de-identified and participant names replaced with pseudonyms. Transcripts and open ended survey question responses were read line by line in close detail, with notes added to capture relevant first-order concepts or codes. Through a process of discussion and decision-making, the researchers grouped first-order concepts where commonalities were identified to create concise, overarching or higher-order codes. This process allowed for defining and refining codes and consultation with the stakeholder advisory group on which data should be included within each code. Having formulated the coding framework, transcripts, open-ended survey questions and photographs with their participant description were imported into NVivo software to facilitate the organization of the qualitative data into relevant codes. Once coding was complete, each of the coded sections was summarized within a coding summary, which further helped to identify commonalities and facilitated theme identification across the data set. Members of our stakeholder advisory group were involved in the development of codes and themes and read and provided comment on the interpretation and reporting of the data. The analysis was revised to incorporate feedback on language and interpretation.

**Figure 1:**
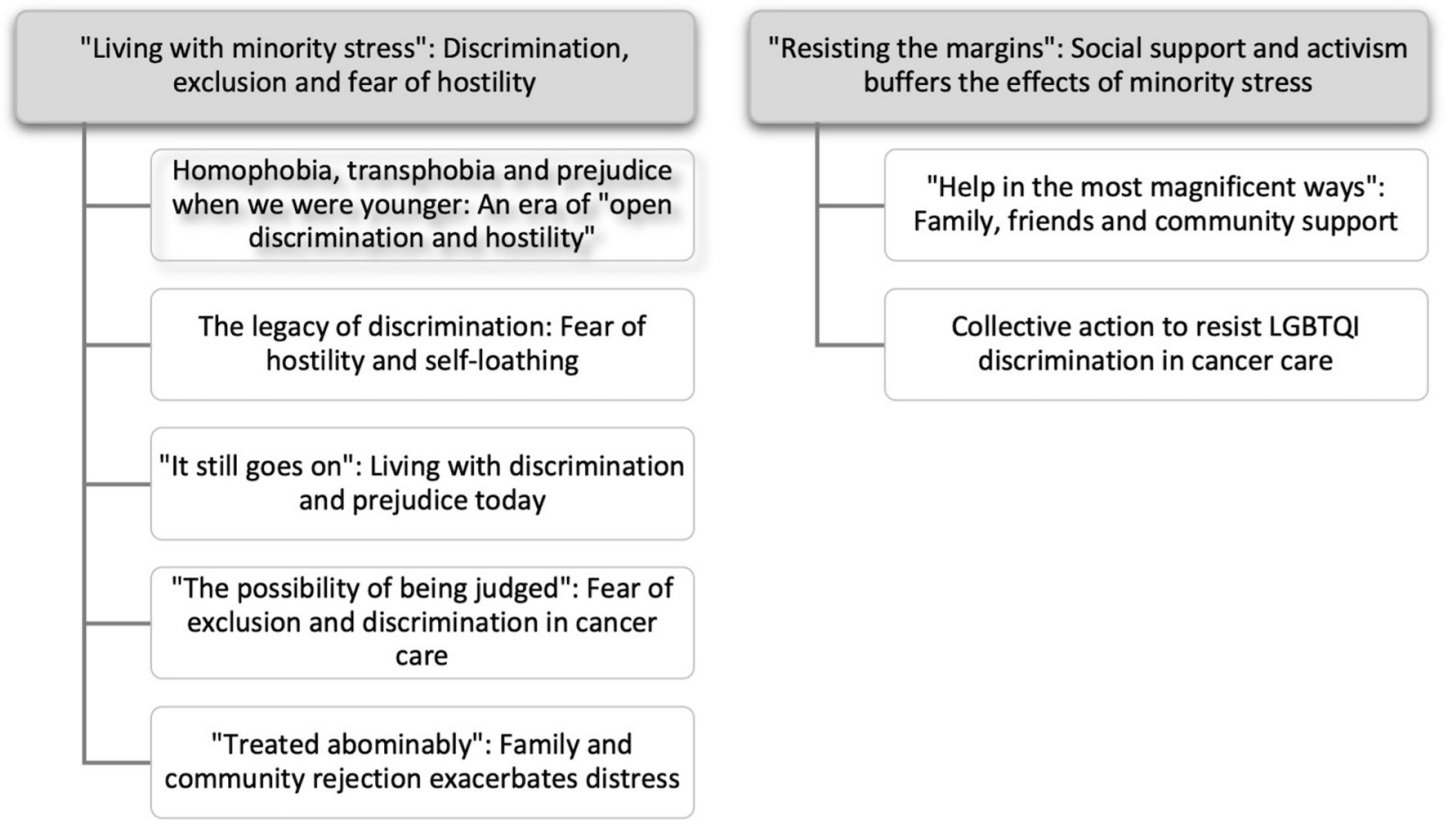
Thematic map.

**Figure 2:**
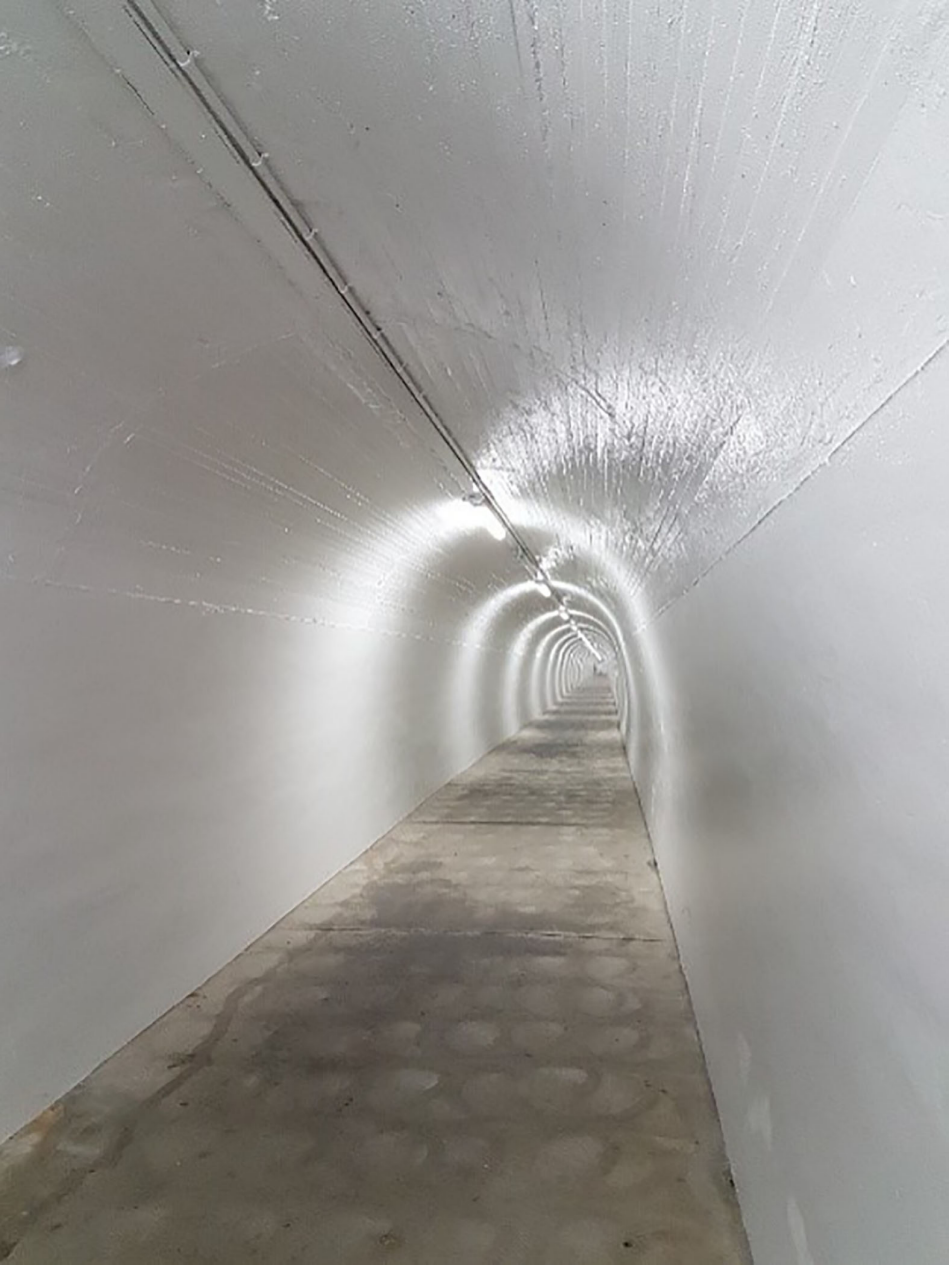
Interiority landmines cycle. In this tunnel, it's mostly safe but it’s like walking in an area that has landmines. You can walk through fine for most of the time and most of the people that you meet will be professional and inclusive. But you’re always cautious. You don't know if you’re going to step on a landmine. So you have to walk gingerly. This is what it means to navigate the health system as a lesbian woman. [Ellen, 36, lesbian, gynecological cancer]

**Figure 3:**
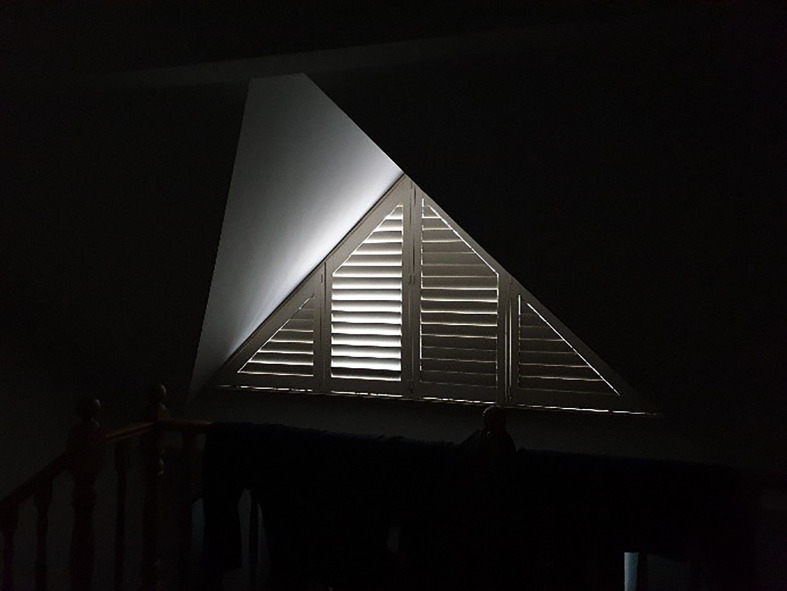
Shutters. When you’re going through cancer treatment, and you've had your diagnosis and everything, it's really important to choose who to let in. Most of the people I spent quality time with were people who I’d intentionally chosen to let into my life. [Mary, 54. Lesbian, breast cancer].

**Figure 4:**
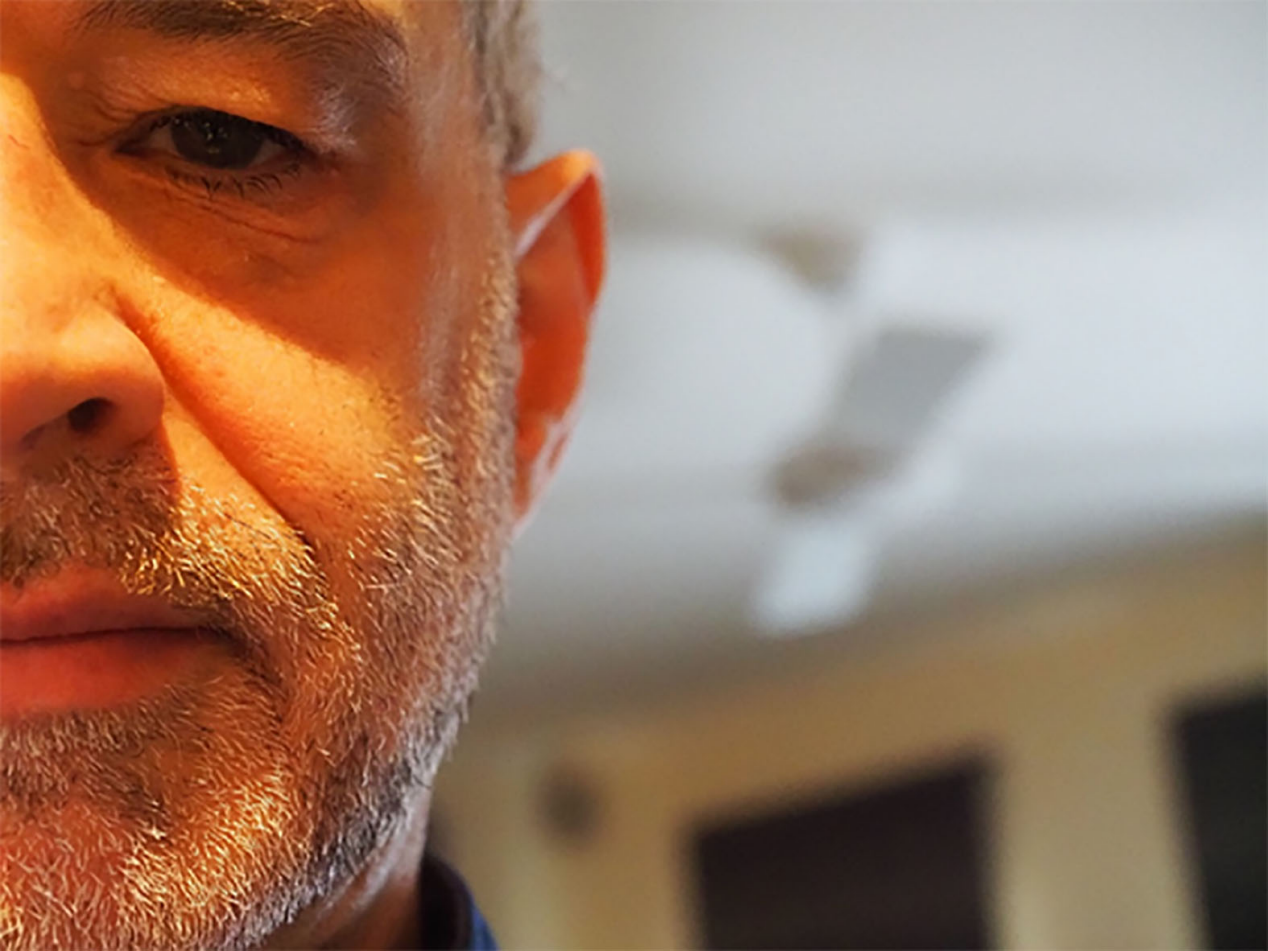
My husband. This is my husband. He, of course, was my main support. As the most important person in my life by way of- well, lots things, but way of support through this. He looked after me in that period no questions asked, no ifs or buts. He took on that role of caring for me. [Rodney. 57, gay man, skin cancer]

**Figure 5:**
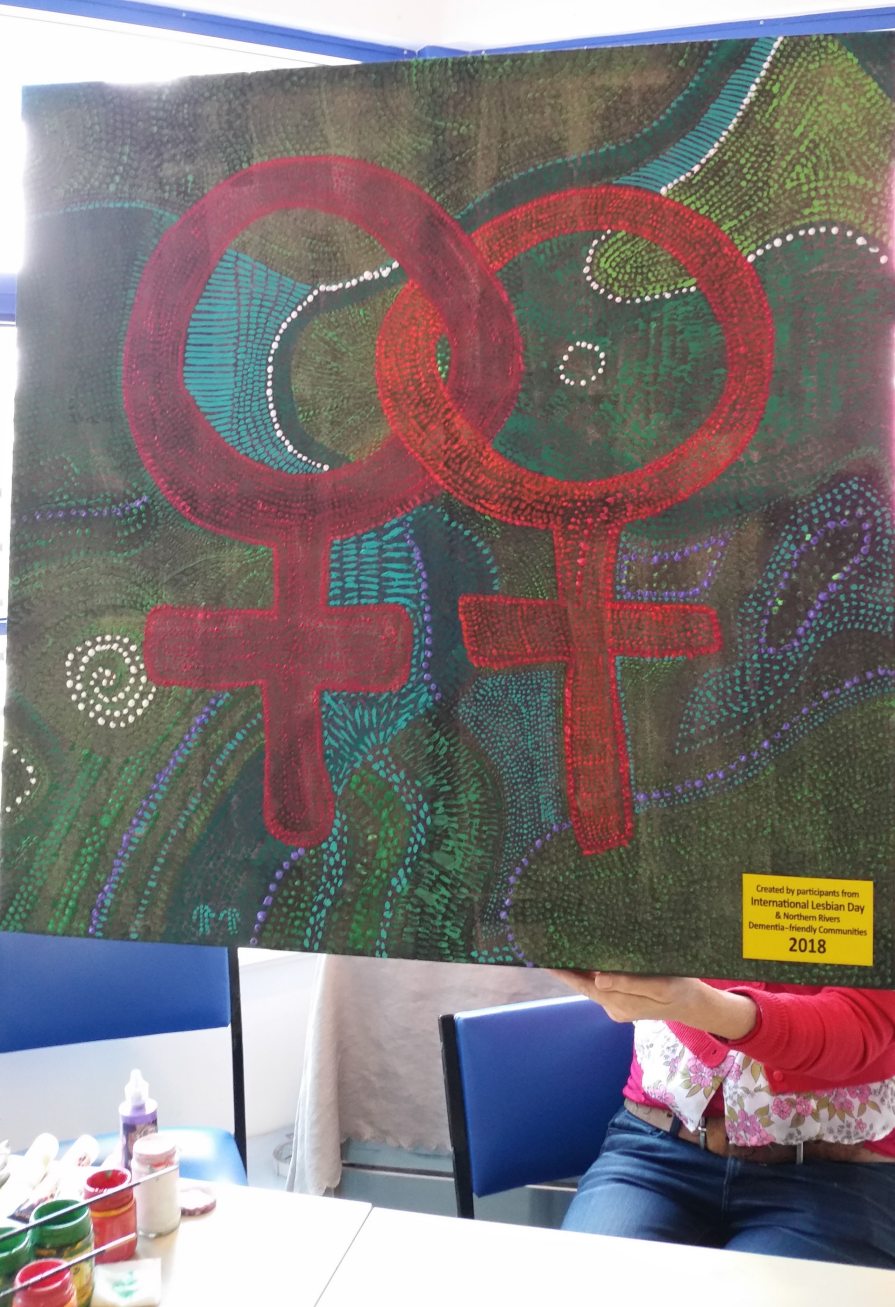
Lesbian support. This painting is about celebrating lesbianism and same-sex relationships. There are about 40 women that come to this group. We all get together and help each other; we’re all working for a common cause – to look after each other as lesbians and to provide support to each other.Maybe there's been a bit of discrimination when you were being treated or some of the nurses were a bit snarky and you could tell they weren't comfortable having your partner in the room, you can talk about all of that with these women because at some point they'd all been there and they probably all experienced it. [Paulette, 67, lesbian, colorectal]

**Figure 6:**
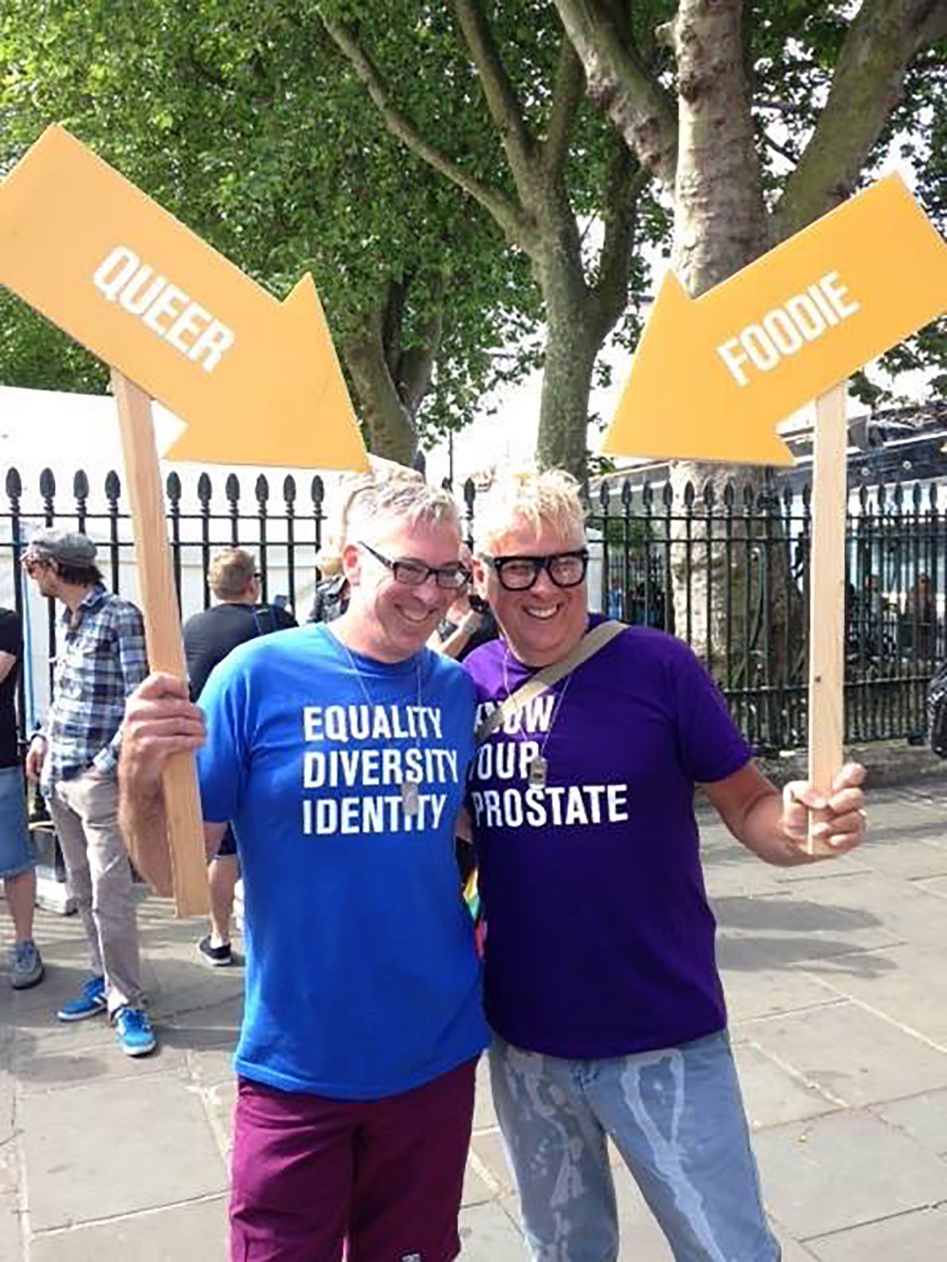
LGBTQ+ activism. Here we are at a pride march. We all had t-shirts and arrows saying different things about our sexualities, genders and interests. Showing that we as a community are completely diverse. I also wore a t-shirt that said, “know your prostate” and as we walked along, I handed out pamphlets about a prostate cancer support group for LGBT people. It felt really important to me to be involved in this activism around sexuality and prostate cancer. [Ryan, 60, gay, prostate cancer]

In the presentation of results, LGBTQI patient and carer participants are identified by pseudonyms (for interviewees) or the word “survey”, with demographic details of age, sexual and gender identity and/or intersex status, and cancer type provided for longer quotes. For readability, demographic details for short quotations are provided in **Supplementary Table 1**, with a longer version of the quotation identified by superscript numerical indicators in the text. Photographs (**Figures 1**–**6**) are accompanied by a brief summary in the words of the participant.

## 3 Results

Two primary themes were identified, each with sub-themes, see **Figure 1**. The first primary theme, “Living with minority stress: Discrimination, exclusion and fear of hostility” describes the cumulative and ongoing experiences of prejudice, discrimination and exclusion which shaped participants’ feelings of safety during cancer treatment, survivorship and care, including impacts on interactions with HCPs and support networks. The second primary theme, “Resisting the margins: Social support and activism buffer the effects of minority stress” documents the role of social support in buffering the negative effects of minority stress in the context of cancer and demonstrates the agency and resistance of LGBTQI patients and carers to discrimination and marginalization during cancer care. The results describe participant experiences of violence, abuse and discrimination; some readers may find these accounts distressing.

### 3.1 Living With Minority Stress: Discrimination, Exclusion, and Fear of Hostility

#### 3.1.1 Homophobia, Transphobia and Prejudice When We Were Younger: An Era of “Open Discrimination and Hostility”

Across participant accounts there was widespread evidence of minority stress in terms of experience of distal stressors. As reported previously, 83.6% of participants reported discrimination in their lives in general because of being LGBTQI (53). In the qualitative analysis, this was described as experiences of stigma, exclusion, hostility and violence. These experiences of minority stress were a legacy that impacted upon LGBTQI patients and carers as they navigated cancer survivorship and care, in relation to feelings of trust and safety.

Participants recounted “a lot of open discrimination and hostility”^1^ and “traumas in our backgrounds”^2^, such as being “bullied at school for being gay”^3,^ including by other children, “parents and teachers”^4^; “bullied for being effeminate”^5^; “verbally abused” and “assaulted”^6^, or “gay bashed”^7^. Discrimination and abuse were perpetrated everywhere, from “family to society”^8^. For example, a 20-year-old lesbian survey participant who was caring for her father who had head/neck cancer said:

My mother and one of my brothers would make homophobic comments in regard to me. I also attended a religious school for most of my life, where it was taught that being LGBT was a sin.

Claire, a 66-year-old lesbian, whose partner had ovarian cancer, said “You couldn’t even be a schoolteacher back in those days”. Discrimination was also inclusive of institutional violence, as a 67-year-old lesbian survey participant with breast cancer commented, “I have PTSD from police violence and witnessing police violence against lesbian and gay people and against Indigenous people in the 70s”.

Many older participants lived through an era when “it was illegal to be gay”^9^, with a minority reporting that they lived in countries where homosexuality is still criminalized. For example, Anthony, a 65-year-old gay man, caring for his husband with prostate cancer, said that for the first seven years of his relationship with his now-husband, “We could have gone to jail”. Lachlan, a 75-year-old queer intersex man with bowel cancer, described growing up during the era of criminalization as “pretty hard” because “everyone’s against you.” He said, “You had to go behind the door sort of thing. You couldn’t tell anyone what you were going through. You had to keep it to yourself.” The widespread persecution of gay men resulted in some participants receiving criminal records for engaging in consensual sex, which contributed to the suicide of friends, and these records that have only recently been expunged. Grace, a 56-year-old bisexual woman with cervical cancer explained the impact of criminalization in her life:

It’s taken me a long time [to embrace my sexual identity] because I grew up in the 70s seeing my friends who were gay being targeted. One guy I worked with took his own life. He had a record because he was involved in a sexual act and that was illegal at that stage. So, seeing all of these things, all of these repercussions, I sort of thought I need to squash the gay side of me.

Some participants commented on the fact that historically, homosexuality and gender dysphoria were “defined as a mental illness”^10^. This cultural discourse positioned sexuality and gender diversity as perversity and a sickness, justifying medical regulation of LGBTQI bodies through practices such as “ECT [Electroconvulsive therapy]” to treat “body dysphoria and sinful sexual desires (attracted to women)”^11^. Scott, a 55-year-old gay trans man with many cancers explained that his partner “was incarcerated in prison for being trans and went through conversion therapy that involved being stripped naked and sitting on the laps of male guards” to “teach you how to be a woman”. A 52-year-old trans woman survey participant with soft tissue cancer said she “failed” conversion therapy, resulting in her being “kicked out onto the street after a particularly bad belting with an electric power cord and a razor strap” and was consequently homeless, “surviving the next 10 years on the streets”. Intersex participants also described medical regulation of their bodies, including “medical violence”^12^ of “forced [sex] assignments” as children, causing “far-reaching consequences”^13^. A 36-year-old queer non-binary/gender fluid survey participant with intersex variations who had medical interventions for cancer said:

The medical community has been nothing but abusive and exploitative regarding my intersex body. I’ve been subjected to medical photography, forced sedation, forced invasive examinations, forced surgical procedures, and lied to about needing surgical procedures under the claim that I had cancerous growths.

During this era of criminalization and pathologization, LGBTQI “people were much more closeted” and “everything was much more hidden”^14^ due to the “threat of losing your job, your housing and definitely family”^15^ if outed in the wrong context. For many participants, coming out as LGBTQI was shrouded in “shame and guilt”^16^ and, for a minority, “being gay was unthinkable” when they were younger^17^. Troy, a 71-year-old gay man with prostate cancer explained that he “didn’t have the courage to come out” and, instead, pursued a heterosexual marriage with children. When he eventually did come out, it “destroyed” and “threatened” his family and was “a very big deal”. These pervasive and cumulative minority stressors experienced by participants over their lives were reference points that contributed to fear, distrust and distress during their cancer journey.

#### 3.1.2 The Legacy of Discrimination: Fear of Hostility and Self-Loathing

There was some evidence of minority stress in terms of proximal stressors, the internalization of anti-LGBTQI sentiments (53), directly impacting upon participants during cancer treatment, survivorship, or as caregivers. Several LGBTQI patients and carers reported that the legacy of prejudice, discrimination, and criminalization, “when we were younger shaped [ … ] responses to things now”^18^, resulting in being “fearful of violence”^19^, “prepared for hostility”^20^ and contributing to “low self-esteem”^5^ and “internalized oppression”^21^. For some, this extended to self-blame for cancer, with Finn, a 56-year-old gay man with throat cancer saying, “I’ve brought this on myself” after a friend joked his cancer resulted from “sucking too much cock”^16^. “Internalized oppression” was also linked to high rates of “smoking, drinking, drug use” in lesbian communities, which are risk factors for cancer^21^. Kai, a 50-year-old, bisexual, trans woman with intersex variations, whose partner had breast cancer, explained that “when you are treated like a leper, you take it out on yourself” and described struggling with “suicide ideation” and “thinking I’m just a burden, I should end it. I should kill myself”. Bernice, a 61-year-old lesbian woman with breast cancer explained the lifelong impact of being the recipient of bullying and marginalization:

When you grow up as a lesbian and the age group that I grew up, your attitudes and the way you deal with the world because of the condemnation, you had to hide things, hide yourself or not be yourself. Or you’d be yourself, but you’ve got to be prepared for hostility or be prepared to shut down, shut down all the external stuff. I think to understand your response to the world, people do need to understand that you are very much affected by that feeling of derision, hatred, whatever, that you’ve blocked out.

Other participants described the long-term and cumulative impact of structural discrimination. For example, Patty, a 71-year-old lesbian woman, whose partner had many cancers, explained that due to LGBTQI and gendered discrimination many lesbian women were excluded from employment opportunities, resulting in their having “less access to good jobs” over the course of their lives. This meant that a number of women were on a “lower-income” and, consequently, “rented for a long time”, negatively affecting their financial scrutiny and putting them “in a very vulnerable position”, when diagnosed with cancer. Patty also noted that many of her peers became isolated as they aged:

I think the other problem is that past discrimination has meant that, probably, some people in my age group and a bit older are hidden away. And they are isolated from family, isolated from the community; and if their partner dies then they’re on their own. And if they’ve got a disability, they don’t get out, so they lose the network. That’s what happens.

Historical discrimination against LGBTQI communities left a legacy of fear for many of these participants, that meant “the anticipation of potential discrimination” was “always there”, contributing to ongoing anxiety and impacting on “health and wellbeing”^22^, irrespective of present-day exposure to such moments. Glenn, a 66-year-old gay man with head/neck cancer said he needed to “very quickly develop a very strong survival code to live in a heterosexual world”, while Anita, a 34-year-old lesbian with uterine cancer, explained that she and her wife:

Used to carry around our marriage certificate everywhere [during cancer care]. We changed our names as soon as we could so if we had to, we could pass off as sisters. We did what we could so that we could make sure that everything was ok. It was a safety concern.

Participants described an “under the skin awareness”^23^ of the potential for prejudice and discrimination during cancer treatment and when caregiving and that they were “judging all the time” so they could “act appropriately to be safe” because “when you’ve feared for your life at different times because of your sexuality, you carry that with you for the rest of your life” ^24^. This legacy of fear created additional layers of stress during cancer diagnosis, treatment and survivorship.

#### 3.1.3 “It Still Goes On”: Living With Discrimination and Prejudice Today

For many participants, prejudice and discrimination were not in the past but were reported as something that “still goes on”^25^, with material and psychological consequences including anxieties about discrimination in cancer care. A 42-year-old lesbian, trans woman, survey participant with skin cancer explained that “being trans”, “discrimination is everywhere. Many people have a deep and visceral hatred of people like me”. The potential for this hatred to culminate in violence was evident in the account of trans participants. A 53-year-old, trans woman, survey participant with soft tissue cancer said:

I’ve been assaulted seriously in the last 24 months, six times. Last year, someone attacked me in my own front yard in daylight. Bathroom use has also been ‘problematic’. I have been verbally abused and physically assaulted on several occasions inside my local shopping center.

Ongoing hostility and prejudice from family of origin was reported by some participants, including being “treated abominably” and in ways that were “totally degrading and frankly inhumane”^26^, because of being LGBTQI. Riley, a 53-year-old lesbian with ovarian cancer, explained that her family “had issues when I came out” causing her to be “estranged from them for a long time because I was an embarrassment”. Catherine, a 61-year-old bisexual woman with vulval cancer, commented, “My actual mother believes that God has told her that me being gay makes me evil and, therefore, it would poison her life to have any contact with me”. Aaron a 32-year old gay man with bowel cancer said;

I think it would be probably a lot easier, like if I wasn’t gay then I would probably have a lot of friends from high school and probably be closer to family members.

Although discrimination could be overt, many participants also reported micro-aggressions pervading all aspects of their lives. Ash, a 40-year-old, bisexual, non-binary person with an unknown primary cancer, said that the relationship with their employer “shifted very quickly” after they were ‘outed’ by a colleague, resulting in increased scrutiny, despite previously “winning awards and getting commendations” at work.

Several participants discussed an “ominous”^27^ increase in recent years of anti-LGBTQI public discourse, including “redneck” comments^27^ and “transphobia in the media”^28^. This engendered fear that the conservative minority would legislate discrimination against and erode LGBTQI rights as “a revenge tactic for ‘you gays and lesbians who got your way’” in relation to marriage equality^27^. As Raymond, a 55-year–old gay man with prostate cancer, commented, “What a lot of us rainbowians, rainbow community people, want is just for the right-wing people to not ruin how things have progressed over the years”. Anti-LGBTQI discourse was experienced by participants as “incredibly tough”^29^ and “very stressful when you are sick [with cancer] and having to listen to all that”^30^.

#### 3.1.4 “The Possibility of Being Judged”: Fear of Exclusion and Discrimination in Cancer Care

Fear of discrimination due to anti-LGBTQI public discourse had material consequences for participants’ engagement in cancer healthcare, illustrated well in the photograph ‘interiority landmine’ (**Figure 2**). Participants described concerns that transphobic and homophobic public discourses would “exacerbate people [HCPs] who are tunnel-visioned and hateful”^31^, thereby legitimizing discrimination. For example, Elsie, a 55-year-old lesbian with lung cancer, said she was concerned that the Australian Religious Discrimination Bill (currently lapsed in federal parliament) would “give doctors an opportunity to say, ‘Well, it’s against my religion to treat you, so I’m not going to treat you’”, causing her to be “nervous” about “going to see a doctor that I’d never met before” due to “the possibility of being judged”. Raymond, a 55-year-old gay man with prostate cancer, worried that prejudiced HCPs “might withhold things or they’ll do indirect discrimination. They’ll do it in sneaky ways, so it won’t look like it’s being discriminatory”.

Participants described feeling “trepidation”^32^ and being “wary”^33^ when deciding if to disclose being LGBTQI to their cancer HCPs, “especially at the time [when] some doctors in Australia were openly against marriage equality”^34^. Some participants said they would “avoid” disclosing being LGBTQI “with medical professional[s] except where it’s clinically relevant”^35^. Oscar, a 27-year-old, gay man with lymphoma said:

I don’t want them [HCP] to know I’m gay because I don’t want them to treat me different. If they realize I’m gay … if they’re religious, are they going to have less motivation to treat me, cure me?

Others said, “I do not refer to it, even though it might be relevant”^36^. Some participants described avoiding medical care because of perceived discrimination:

The world we live in - read the news - affects our health. The more we know we are hated and feared (religious freedoms act; trans hate; The Australian [a News Corporation newspaper]), the less likely we are to access care or feel safe when we do. One of my specialists was showing me an app on her phone and it was surrounded by Christian apps and Bible apps, and I was instantly terrified and will not go back. (Survey, 40, intersex, queer, medical intervention)

For many participants, discrimination in cancer care or as part of caregiving was a lived reality, with 33% of participants reporting discrimination in cancer care, as reported previously (53). Participants reported exclusion of same-sex partners and inadequate care. Ryan, a 60-year-old gay man with prostate cancer, said “I didn’t feel supported. I didn’t feel like my husband was included”, and a 57-year-old, asexual, trans man, survey participant, who had medical interventions for cancer risk and was caring for his partner with many cancers, said,

Discrimination has been around if we are really in a relationship. We have had many times where me, or my partner, have been refused entry into the ICU saying, “only family may visit!” It is extremely distressing to be denied the support of your partner.

Several trans patients described micro-aggressions and exclusion in having to navigate healthcare settings, which were underpinned by dominant discourses of a binary conception of gender. Trans patients and carers reported being “misgendered”^37^ by HCPs, having to receive treatment in cancer spaces that did not align with their gender identity, or concealing their trans status to pass as “cis”, causing negative psychological consequences. A 38-year-old, queer, non-binary person, who stopped taking hormones to present as more feminine when caring for their grandmother who was in hospital with bowel cancer, explained that “to feel that I had to pretend to be someone else was upsetting and stressful”.

Other patients described religious prejudice enacted by HCPs: “One of the nurses was quite religious and said that she would pray for her … because she was gay, not because she had cancer” [Mary, 54, lesbian, breast]:

He [healthcare professional] clearly read me as a lesbian and he was dismissive of me as a person. It kind of felt like I was being treated like a lesser person. And that judgment was based on his belief system. Early on in the conversation, he identified as a religious person and then I kind of pieced together why he was being rude and paternalistic and judgmental. [Jasper, 50, queer woman, breast]

Exclusion was also experienced at the intersections of cultural background, gender and sexuality, evident in cancer information resources, as a 38-year-old, non-binary/gender fluid lesbian, queer survey participant with many cancers said: “It’s all really white, and white Australian. My partners have not always been white, and they felt actively excluded from all of the materials I brought home for their sexuality, gender and race”.

A number of participants described experiencing prejudice from “other patients”, including a 58-year-old survey participant caring for her lesbian friend with cervical cancer, who said, “My friend was in a waiting room and there was a segment about LGBTIQ+ rights, and a couple sitting next to her said some terrible things about LGBTIQ+ people. It was very upsetting for her”.

#### 3.1.5 “Treated Abominably”: Family and Community Rejection Exacerbates Distress

Many participants reported rejection from their families of origin, local communities or LGBTQI sub-groups, adding to minority stress and impacting on feelings of connection and support during cancer treatment and survivorship and caregiving.

Several participants reported anti-LGBTQI prejudice and exclusion from their families of origin. Lucinda, a 59-year-old queer woman whose partner had ovarian cancer, recounted telling her partner’s niece, “It’s your fault that she [partner] died because you don’t believe in God”. Some participants “choose not to interact with”^38^ family members who were not accepting of them being LGBTQI, including a 22-year-old, bisexual, survey participant caring for her mother with breast cancer, who said that “cancer showed me that my family is not just poor support, but is a burden, so I have distanced myself from them to take better care of myself.” Being discerning about support during cancer was illustrate by the photograph “Shutters”, **Figure 3**.

Others had to navigate relationships with hostile relatives, either to give or receive care during cancer. Kai, a 59-year-old, bisexual, trans, intersex woman whose partner had breast cancer, explained the impact of family prejudice on caring for her partner with cancer:

I have had to care for my partner from afar as some of my family have rejected me for being trans. This has been a nightmare. We had the discussion after diagnosis as to what life meant and we both agreed that it is important to be truly happy. I then got the courage up to transition, with her full support, and now her family and some of my own have rounded on me for this. So, not only was she fighting cancer, she was fighting prejudice and such as well. It’s put a whole lot of extra layers of extra stress on her, on her health.

Other participants said they concealed their sexuality or gender identities due to a “realistic concern”^39^ of being disowned and fear of judgement, or due to safety concerns. However, this sometimes meant that their partners were unable to access support during cancer, as a 26-year-old gay male survey participant, caring for his partner who had prostate cancer, commented:

My partner is an Asian Muslim and has to hide his sexuality totally from his family in fear of his psychological wellbeing and, possibly, his life too. Because of this, I have no one to share my thoughts and feelings with. During chemotherapy, I was privately scared that he would die, and I felt I could not share that with him or my own family and friends because of the consequences.

For a minority of participants, lack of family support during cancer placed additional pressure and stress on intimate relationships. A number of participants described their relationships ending or becoming abusive:

My partner of four years broke up with me after a diagnosis. She did not feel she had the support around her to support me or to see me die. I was in a six–week period between two major surgeries and two different diagnoses. The mental health impacts during such a time were devastating. (Survey, 37, queer, non-binary/gender-fluid, medical intervention)

Lack of family support also exacerbated economic vulnerability during cancer treatment as “we don’t have security from our parents as some people in our generation might because we’re gay”^40^. This vulnerability accumulated for some, mostly younger participants, to create housing insecurity and homelessness. For example, Alex, a 35-year-old gay, non-binary person with testicular cancer did all his radiation treatment “with no firm place where I was living, no place to call home”. Exclusion and isolation from family were described as “emotionally distressing”^41^ and meant that some participants “don’t particularly have anyone to rely on for much care or help, so it’s just me dealing with it when I need to go to appointments, [or] be in hospital”^42^. A 57-year-old, asexual, trans man, survey participant who had medical interventions commented, “There is very little support out there when you don’t have family of origin supporting you. General services, like hospital social workers or council home-help range from totally ignorant to outright prejudice”.

A number of participants said they felt unwelcome and alone in their local communities, particularly those living in conservative, religious and regional/rural communities. For example, an 18-year-old, bisexual, survey participant, caring for her father with lung cancer, said, “I don’t feel welcome anywhere as I live in a very Bible belt area”. Carter, a 20-year-old gay man with leukemia, living in a regional area, said:

There just aren’t many people, in general, around that area. You know, you could go into town and there’ll be a few people, but none of them would be gay. So, meeting other gay people, that wasn’t really an option, where I lived.

A 34-year-old, queer, non-binary/gender fluid, survey participant, caring for their partner with breast cancer, said, “I constantly looked for groups of people like me, but I never found anyone. I assume the few others out there, who are the same age and community as me, were just as isolated and stressed and also couldn’t make the contact with anyone to get peer support”. At the same time, cancer support groups including “online communities” were described as “very heteronormative”. A 42-year-old, queer, non-binary/gender fluid, survey participant with breast cancer said that accessing support through these pathways left them feeling “more isolated and ‘different’ than I have ever felt”.

For some participants, particularly trans and bisexual participants, LGBTQI communities were experienced as unwelcome, hostile, and discriminatory. A 39-year-old bisexual, woman, survey participant with cervical cancer said that “mainstream gay and lesbian culture is hostile to bisexuals” and a 49-year-old trans man survey participant with ovarian cancer said that “people assume the LGBTQI community is a source of support … it often isn’t. Transgender people face discrimination from within LGBTQI communities”. Others described LGBTQI communities as “superficial” and “lacking in compassion and empathy”, with “no old-fashioned caring”, just “strangers looking out for their own needs”^43^. This perspective was evident in the accounts of older gay men, who contrasted their present-day experiences of exclusion with the culture of community caregiving experienced during the HIV epidemic of the 1980s and 90s. These participants said that, despite being at the forefront of fighting for rights earlier in their lives, they now felt “old” and “invisible” in contemporary LGBTQI spaces^44^.

### 3.2 Resisting the Margins: Social Support and Activism Buffer the Effects of Minority Stress

#### 3.2.1 “Help in the Most Magnificent Ways”: Family, Friends, and Community Support

Social support played an important role in buffering the negative impacts of minority stress and in helping LGBTQI participants deal with cancer (53), with 78% of participants reporting strong social support (53). This was described as access to “a very large support network”^45^ of “family, friends, acquaintances and work colleagues”, who offered “unconditional and full support”^46^. This was experienced as “nourishing”^47^ and “revealed new depths of connection, love and respect”^48^. For Carol, a 40-year-old gay woman with breast cancer, having a reliable, supportive network meant that she could focus on “just being sick”, She described feeling that she could “just float around because there were really big groups of people who were looking after my [wife] and my kids”.

For many patients, intimate partners were their “main support”, illustrated by the photograph ‘My husband’, **Figure 4**. Partners often “paved the way”^49^ and helped navigate cis-heteronormativity and discrimination in cancer care. It was commonly reported that “going on hospital visits and that sort of thing together”^50^ with same-gender partners helped disclose to HCPs, get information, and advocate for needs. Anita, a 34-year-old lesbian with uterine cancer, said “She [my partner] was the best supporter, helped me advocate for myself and keep everything straight and figure out what I supposed to – what I wanted to say, help me think through stuff but also support me in my own decisions”. Barry, a 56-year-old gay man whose husband had lung cancer, said that navigating cancer together had strengthened their relationship:

It’s really shown what an incredibly resilient couple we are. It’s really brought forward all of the beautiful things that have underpinned our relationship over the last 24 years. They suddenly light up large in big print. The reasons that we have worked so well as a couple.

Participants also reported strong support from family and friends (53). This family support was positioned as crucial to wellbeing, as “people survive longer when they’ve got things like cancer if they’ve got family support”^51^. A 40-year-old, queer, non-binary/gender fluid, survey participant caring for their partner with breast cancer. said that “my parents and in-laws [ … ] would help us in a heartbeat when we asked for it” and an 80-year-old lesbian survey participant, whose wife had kidney cancer, told us: “My family rallied around my wife as she battled cancer and then supported me after she died”. Participants often described being “grateful”^52^ for support from their families, and this was seen as a reflection of acceptance of being LGBTQI. Sandra, a 69-year-old lesbian, who had cared for her partner with breast cancer, said she felt “fortunate” because:

My [partner’s] parents could have come into step at any time and pushed me out. They didn’t. They didn’t stop me going into Emergency. They always deferred to me and at the funeral made sure I was included. I think they wished she [partner] wasn’t a lesbian because it’s not natural to them. But they never, ever rejected me.

Other participants said that cancer “increased my family’s respect and perseverance of me”^53^, resulting in them having “grown closer”^54^.

For many participants, support from other LGBTQI people, described as chosen family, was crucial during cancer. Chosen family served to ameliorate strained or estranged relationships with the family of origin and “were the ones that were there for me”^55^, providing support “in the most magnificent ways”^56^. Paulette, a 67-year–old lesbian woman with colorectal cancer, explained:

My [natal] family wasn’t terribly supportive at that time or since, basically. I think that’s why I have so much emphasis on my chosen family, which is a lesbian community. They’ve kind of replaced my biological family.

Chosen family created an important sense of belonging and reinforced LGBTQI identities, particularly among older lesbians. A 78-year-old lesbian survey participant said, “Being a lesbian carer for my lesbian lover was a very empowering and emotional time because the radical lesbian feminist community we were part of were very supportive”. She explained that it was “an opportunity for us all to pull together in quite extraordinary ways”. Being part of a community where everyone “shares the load of caring for one another”^57^ was regarded as protective against isolation and loneliness in the context of living with cancer. As a 75-year-old lesbian survey participant caring for her partner with breast cancer commented, “Without the love of my lesbian friends, I would feel very alone”. A 55-year-old lesbian survey participant with ovarian cancer said, “Almost every weekend since diagnosis, we’ve had someone visit. I’ve felt so lucky, so loved, like all my birthdays come at once. And that’s made everything easier”. Alice, a 48-year-old lesbian caring for her ex-partner with breast cancer, explained that support from her chosen family was often all-encompassing due to shared experiences of marginalization:

When you think about the queer community compared to the straight community, it just seems to be there’s a sense of all-hands on deck and everybody bringing what they can to a situation, and I think that really comes from needing chosen family to navigate your way, through being othered so much. The sense of community and chosen family is very, very strong.

Some participants described receiving support during cancer from unexpected sources, including work colleagues. A 40-year-old lesbian survey participant whose partner had uterine cancer, said that she was usually quite private at work, but that cancer was a catalyst for disclosing both her LGBTQI status and partner’s cancer diagnosis. When she did, it enabled her to engage more authentically with colleagues, access support and negotiate flexible work arrangements: “I had to come out to a lot of colleagues during this process. However, they were all very supportive, which made it easier.” Other participants living in regional areas said that, despite the potential for social isolation due to there being few other visible LGBTQI people, they received “nothing but love and care” from within their local communities during cancer. This experience was often discussed by older participants who were “unapologetically out” as LGBTQI in their communities. Barry, a 56-year-old gay man whose husband had lung cancer said:

We made the decision before we moved into our regional town to go in from the outset as an out, gay, mixed-race couple, which was a little bit brave. Weirdly enough, we’ve been wrapped with more love than we expected to be in the country. And sometimes, it’s actually been quite beautiful. Not once have I had anyone come up and say, “I’m sorry to hear about your friend”. Everyone has said, “I’m really sorry about your husband”.

For many participants, pets were an important source of companionship and support that helped ameliorate loneliness and regulate emotional distress including anxiety and grief. Pets were described as “the most incredible companion”^58^ that was “always by my side”^59^ offering “constant company”^60^. Leonard said his dog had “given me more support over the last, nearly seven years than anything else in my life”. For Neal, a 68-year-old gay man with prostate cancer whose partner had died, his dog played an important role during long nights when he was struggling with anxiety and loneliness.

#### 3.2.2 Collective Action to Resist LGBTQI Discrimination and Exclusion in Cancer Care

Collective activism, combined with social support for others, was described by participants as having served to buffer the impact of minority stress. Participants discussed positive experiences in LGBTQI-specific support groups, shared experience in the context of both cancer and LGBTQI community, illustrated in the photograph “Lesbian Support” (**Figure 5**). A number of participants said that “having this cancer has made me realize I will never be put in the closet again”^61^, motivating them to become “politically involved”^62^ and “very vocal about my experiences”^63^, to advocate for LGBTQI-specific cancer information and support for their communities. A 30-year-old intersex lesbian who had medical interventions for cancer risk said that after being “closeted for seven years as being intersex due to doctors telling my parents and I to keep my diagnosis a secret from everyone I knew, I got angry and shared my story with a local newspaper and now advocate for youth internationally”. A 49-year-old trans man survey participant with ovarian cancer said that they were now “providing presentations to colleagues with the aim to increase understanding of working with transgender cancer patients”. For other participants, activism involved “coming out”^64^ within cancer care as a way to “challenge the systems that I come in touch with”^65^:

I came out to so many people in the hospital – staff and other ‘cancer families’ – often for the reason of increasing visibility of rainbow families in the hope that this would reduce homophobia, and increase people’s awareness of rainbow families and just how similar/normal we are when compared with families headed by a heterosexual couple or heterosexual single parent (Survey, parent, 47, lesbian, leukemia)

Several participants set up “support groups”^66,67,68^ in their local area offering connections to other LGBTQI people with cancer. This included one person who started “a charity to support other LGBTIQ cancer patients”^69^ and another who had “produced a booklet”^70^ to share information about cancer in LGBTQI communities including cancer screening and cancer survivorship. Participants explained that their LGBTQI cancer advocacy was motivated by the desire to “use our experiences to be of help”^68^ and “because they [LGBTQI cancer information/services] weren’t there for me so I want to make sure they can be there for someone else”^69^. Being involved in LGBTQI cancer advocacy was also discussed as affirming identity and helped to build community connection, illustrated in the photograph “Know your prostate!”, **Figure 6**. Dylan, a 35-year-old gay non-binary/gender-fluid person with leukemia explained that it “brought me around many LGBT+ people where I can express who I am”. Paulette, a 67-year-old lesbian with colorectal cancer, said that being involved in LGBTQI cancer support helped her to feel “a bit more part of the community and to be with people who don’t question who you are and why you are the way you are and that type of stuff” and experience she described as “like coming home”.

## 4 Discussion

The aim of this paper is to examine subjective experiences of minority stress and social support for LGBTQI people with cancer in intersecting sexual and gender identities, intersex status, age, and tumor types. Our findings provide insight into the chronic and cumulative nature of minority stress and multiple systems of oppression that have shaped historical and present-day anti-LGBTQI prejudice, discrimination, family rejection and exclusion from communities. These experiences have exacerbated fear and distress during cancer survivorship and caregiving. However, social support and advocacy did ameliorate minority stress for LGBTQI patients and carers, and for some, affirmed their identities. That said, some participants lacked social support, or experienced rejection from those whom they turned to for support, exacerbating their distress.

Our findings confirm previous reports that LGBTQI people with cancer and their carers experience unique stressors during their lives, which serve to exacerbate distress in cancer survivorship (3, 6). Many of the older participants became adults during an era when homosexuality was criminalized and considered a mental illness, resulting in societal and institutional discriminations, stigma and violence. Lack of LGBTQI legislative protections during that era legitimized abuse, allowing bullying and hate crimes to go unprosecuted (67). So-called reparative practices, such as LGBTQ conversion therapies, were administered under the guise of a therapeutic framework (68) and “normalizing” medical interventions were conducted as routine practice on infants with intersex variations (69). Recently, LGBTQ conversion therapies were publicly acknowledged to be cruel, degrading forms of torture (68) and there is growing acknowledgement that sex assignments conducted in infancy on individuals with an intersex variation violate bodily autonomy and deny human rights (69, 70). The long-lasting proximal stress caused by these traumatic experiences, such as distrust and fear of prejudice, concealed identities and internalized stigma (23), were evident in the accounts of participants in the present study, sometimes years after the events had taken place. This creates a vulnerability that impacts the experience of cancer and cancer care (3, 27, 53). Such minority stress has also been demonstrated to cause long-lasting negative psychological consequences (71) and is a risk factor for premature cognitive decline (72).

In liberal democracies, LGBTQI people experience greater equality today, including legislative protections in their everyday lives. Many countries have decriminalized same-sex relationships, passed anti-discrimination legislation, and have put in place protections against hate speech and hate crimes (73). However, there are continued political and legislative efforts to prevent further equity and to roll back established LGBTQI rights (44, 45). Anti-LGBTQI legislative processes and political campaigns are demonstrated to affect LGBTQI people’s mental health adversely (74, 75), causing higher levels of stress, depression and anxiety, and increased exposure to harassment and discrimination (76). The “pitting” (73) of LGBTQI rights against other human rights, such as religious freedoms (45), has meant that LGBTQI people continue to live in hostile and discriminatory environments that contribute to internalized, health-eroding stressors, including distress and fear during cancer survivorship and care (27).

Our findings demonstrated the intersection of identities in the experience of minority stress, providing insight into the quantitative findings of the Out with Cancer Study, where trans, intersex and AYA patients and carers reported higher rates of minority stress (53, 55). Our findings confirm previous reports that trans people experience high rates of verbal, physical and sexual violence (63, 77) and discrimination in healthcare (78–80). This is compounded by anti-transgender discourse that appears in the news, with negative implications for feelings of safety and wellbeing (47, 48). People with intersex variations also face numerous societal discriminations and hostility (70), as well as mistreatment in medical settings, including violations to bodily autonomy that deny human rights, leading to distress (69). Younger LGBTQI people can experience bullying and social exclusion, at a time when many are exploring their sexuality and identities (81). Young people have also been the target of recent transphobic media reports, with negative consequences for mental health (47). The impacts of minority stress were also evident in LGBTQI patient and carer accounts of discrimination and or fear of discrimination in cancer care. LGBTQI patients who feared discrimination, or had previous negative experiences in healthcare, reported poorer mental health outcomes, greater distress and unmet needs (6, 53, 82) and may avoid care (83). This results in concealment of LGBTQI identities from HCPs (80), as was evident in the accounts of some participants in the present study. Whilst concealment of LGBTQI identity can be a self-protective mechanism (80), it is associated with invisibility (6, 84), regret (82), unmet needs (6, 8), and can compound the stress of cancer and lead to poor psychological outcomes (85).

Our findings confirmed and extended previous research that social support can ameliorate the negative impacts of minority stress in LGBTQI populations (3) and is protective against distress (86). Cancer caregivers in the non-LGBTQI community are typically intimate partners (15). For many LGBTQI people, chosen family, including partners, friends and other LGBTQI people, provide support and connection (24, 87). Support from chosen family affirmed LGBTQI identities and relationships and offered group solidarity, confirming previous reports (24). The culture of mutual caregiving within LGBTQI communities is partly a cultural legacy of the HIV/AIDS era, whereby caregiving was “thrust” (88) upon gay and bisexual communities as a result of discrimination in healthcare and family rejection (89). There is also a long history of feminist lesbian community activism and connectiveness (90), and more recently, trans community visibility and mutual support (91). However, for a minority of LGBTQI people with cancer, trans and bisexual participants in particular, experiences of in-group microaggressions meant that broader LGBTQI communities were experienced as exclusionary, hostile and unsafe (92), increasing vulnerability to social isolation and distress. This further highlights the way in which intersecting identities can lead to vulnerability, precluding the buffering effect of social support for some LGBTQI people with cancer. It is important to note we found no discussion of microaggressions within LGBTQI cancer support groups.

For younger LGBTQI participants, support and acceptance from family of origin were important to psychological wellbeing, as reported in previous research (49, 93). However, the ameliorating role of social support was dependent on families affirming LGBTQI identities; support that was hostile or required LGBTQI people to conceal their identities added to minority stress. Younger LGBTQI people with cancer and carers living outside of metropolitan hubs often lacked LGBTQI community support due to the absence of other LGBTQI people where they lived (94). Young people who are discovering or exploring their identities, and who do not have strong connections to LGBTQI chosen family, may also lack affirming social support (81). These findings highlight the ways in which LGBTQI identity and age intersect to create a position of marginalization for adolescents and younger adults with cancer.

Our findings also demonstrated that LGBTQI cancer carers experience minority stress, which can be ameliorated by social support, as is the case for LGBTQI people with cancer. Support for LGBTQI caregivers is associated with better mental health (95), reinforcing the need for LGBTQI carers to be included in cancer support (6). Due to the pervasive impacts of cis-heteronormativity in cancer care and support groups (27), LGBTQI carers may be uncomfortable accessing services and support available to non-LGBTQI carers (13). It is essential that the needs and experiences of carers are included in future LGBTQI cancer research, and in programs of LGBTQI cancer care.

LGBTQI patients and carers were not passive recipients of discrimination and exclusion in cancer, demonstrating agency and resistance through collective action and advocacy. The provision of support to other LGBTQI people with cancer through support groups or other forms of activism served to affirm identities in the face of minority stress among participants of all ages. LGBTQI communities have a history of mobilizing to affect change and improve LGBTQI rights, which has had the effect of building community and solidarity (90, 91). However, the requirement for people experiencing adversity to address health inequities within their communities places an undue burden on marginalized individuals and communities, highlighting the need for systemic changes to reduce minority stress in cancer survivorship and care.

## 5 Conclusion

In conclusion, our findings demonstrated that LGBTQI people have unique socio-political histories and present-day experiences that have created a legacy of distrust and fear and may contribute to distress and unmet needs in cancer survivorship and care (6). For LGBTQI individuals, minority stress compounds the impact of other stressors associated with cancer, including fear of cancer recurrence, uncertainty of treatment outcome, co-morbidity and symptomatology (3, 96, 97). Social support serves to buffer the impact of minority stress (3, 49), and is widely recognized to be associated with better quality of life and psychological wellbeing for people with cancer (15), including LGBTQI cancer populations (5, 51, 98).

Our analysis across the mutually constitutive intersections of gender, sexuality, intersex status, cultural background and age identified intersectional differences in the nature and impact of minority stress and social support for LGBTQI people with cancer and their carers. The impacts of minority stress are compounded for LGBTQI people who occupy multiple marginalized positions (99), with those who are trans or gender diverse, intersex and/or of younger age being particularly vulnerable (53). However, strong social support serves to buffer minority stress, with both family of origin and chosen family, including partners and other LGBTQI people, serving as vital and empowering sources of support for LGBTQI patients and carers.

Our findings have implications for LGBTQI cancer care and support services. Environments in which LGBTQI patients and carers access care and support during cancer need to be culturally safe and inclusive (27, 100). This can be achieved through professional education and training, ensuring HCPs are knowledgeable about the potential impacts of historical and present-day minority stressors on cancer survivorship, including higher risks of distress and barriers to social support, particularly for trans, intersex and AYA patients and carers. Awareness of the needs of LGBTQI patients and carers will enable HCPs to adopt inclusive and reflective practices (27, 101). Due to the pervasive impacts of cis-heteronormativity in cancer care and support groups, LGBTQI patients and carers can be uncomfortable accessing services and support for non-LGBTQI people (13), further exacerbating isolation and vulnerability to poor psychological wellbeing (20, 21). Visual indicators of LGBTQI inclusivity, such as pride flags, can signal awareness and safety for LGBTQI patients, as can acknowledgement of sexual and gender identity or intersex status on patient intake forms and presence of anti-discrimination health service policies (100, 102). The inclusion of partners and chosen family in consultations and support services is also essential (27). There is also a need for investment in peer-led initiatives that provide connection and support to LGBTQI people with cancer and carers to help to overcome barriers to social support. Minority stress is pervasive in the lives of many LGBTQI people. We have a duty of care to ensure that the impact of minority stress on wellbeing and on interactions with HCPs is recognized and addressed within professional cancer care.

## Data Availability Statement

The raw data supporting the conclusions of this article will be made available by the authors, without undue reservation.

## Ethics Statement

The study received ethics approval from Western Sydney University Human Research Ethics Committee (ref. no. H12664, with secondary approval from the ACON (formerly the AIDS Council of New South Wales) (ref. no. 2019/09). Written informed consent to participate in this study was provided by the participants’ legal guardian/next of kin. Written informed consent was obtained from the individual(s) for the publication of any potentially identifiable images or data included in this article.

## The Out With Cancer Study Team Members

Chloe Parton^1^, Cristyn Davies^2^, Gary W. Dowsett**^3^**, Katherine Boydell^4^, Fiona McDonald^5^, Antoinette Anazodo^6^, Kerry H. Robinson^7^, Felix Delhomme^8^, Martha Hickey^9^

^1^ School of Health, Te Herenga Waka – Victoria University of Wellington, Wellington, New Zealand

^2^ Specialty of Child and Adolescent Health, Faculty of Medicine and Health, University of Sydney, Australia; and School of Social Sciences and Psychology, Western Sydney University

^3^ Australian Research Centre in Sex, Health and Society, La Trobe University, Melbourne, Australia

^4^Black Dog Institute, University of New South Wales, Sydney, Australia

^5^ Canteen and Faculty of Medicine and Health, The University of Sydney, Sydney, Australia

^6^Kids Cancer Centre, Sydney Children’s Hospital and School of Women’s and Children’s, University of New South Wales, Sydney, Australia

^7^ School of Social Sciences and Translational Health Research Institute, Western Sydney University, Sydney Australia

^8^ACON, Sydney, Australia

^9^Department of Obstetrics and Gynaecology, University of Melbourne and the Royal Women’s Hospital, Melbourne, Australia

## Author Contributions

JU and JP designed the study and prepared the application for funding, in collaboration with The Out with Cancer Study team. The survey was developed by JU, KA, and RP in collaboration with the Out with Cancer Study team, and our stakeholder advisory group. Data were collected by RP and KA. RP conducted qualitative analysis of the data, in collaboration with JU. RP and JU wrote the paper, with critical input from JP, AH, and KA. The Out with Cancer Study Team provided critical commentary on the written paper. All authors approved the final paper.

## Funding

This study was funded by the Australian Research Council Linkage Program grant [LP170100644], the Cancer Council New South Wales, and Prostate Cancer Foundation Australia, with in-kind support provided by LGBTIQ+ Health Australia, ACON, Breast Cancer Network Australia, Sydney Children’s Hospital Network, and Canteen.

## Conflict of Interest

The authors declare that the research was conducted in the absence of any commercial or financial relationships that could be construed as a potential conflict of interest.

## Publisher’s Note

All claims expressed in this article are solely those of the authors and do not necessarily represent those of their affiliated organizations, or those of the publisher, the editors and the reviewers. Any product that may be evaluated in this article, or claim that may be made by its manufacturer, is not guaranteed or endorsed by the publisher.

## Abbreviations

ANZUP, Australian and New Zealand Urogenital and Prostate Cancer Trials Group; AYA, Adolescents and young adults (15-39 years); HCP, Healthcare professional; LGB, Lesbian, gay and bisexual; LGBQ, Lesbian, gay, bisexual and queer; LGBT, Lesbian, gay, bisexual and transgender; LGBTQI, Lesbian, gay, bisexual, transgender, queer and/or intersex; SGM, Sexual and gender minority; Trans, Transgender people, binary and non-binary.

## References

[1] Mitchell AJD Ferguson DW Gill J Paul JB Symonds PP. Depression and Anxiety in Long-Term Cancer Survivors Compared With Spouses and Healthy Controls: A Systematic Review and Meta-Analysis. Lancet Oncol (2013) 14(8):721–32. doi: 10.1016/S1470-2045(13)70244-4 23759376

[2] Ussher JM Perz J Kellett A Chambers S Latini D Davis ID. Health-Related Quality of Life, Psychological Distress, and Sexual Changes Following Prostate Cancer: A Comparison of Gay and Bisexual Men With Heterosexual Men. J Sex Med (2016) 13(3):425–34. doi: 10.1016/j.jsxm.2015.12.026 26853048

[3] Kamen C Mustian KM Jabson JM Boehmer U. Minority Stress, Psychosocial Resources, and Psychological Distress Among Sexual Minority Breast Cancer Survivors. Health Psychol (2017) 36(6):529–37. doi: 10.1037/hea0000465 PMC5444950 28165265

[4] Pratt-Chapman ML Alpert AB Castillo DA. Health Outcomes of Sexual and Gender Minorities After Cancer: A Systematic Review. Systematic Rev (2021) 10(1):1-30. doi: 10.1186/s13643-021-01707-4 PMC8218456 34154645

[5] Desai MJ Gold RS Jones CK Din H Distz AC Shliakhtsitsava K. Mental Health Outcomes in Adolescent and Young Adult Female Cancer Survivors of a Sexual Minority. J Adolesc Young Adult Oncol (2021) 10(2):148–55. doi: 10.1089/jayao.2020.0082 PMC8064930 32730111

[6] Lisy K Peters MDJ Schofield P Jefford M. Experiences and Unmet Needs of Lesbian, Gay, and Bisexual People With Cancer Care: A Systematic Review and Meta-Synthesis. Psycho-Oncology (2018) 27(6):1480–9. doi: 10.1002/pon.4674 29462496

[7] Clarke M Lewin J Lazarakis S Thompson K. Overlooked Minorities: The Intersection of Cancer in Lesbian. Gay Bisexual Transgender and/or Intersex Adolescents Young Adults (2019) 8(5):525–8. 10.1089/jayao.2019.0021 31199707

[8] Quinn GP Sanchez JA Sutton SK Vadaparampil ST Nguyen GT Green BL. Cancer and Lesbian, Gay, Bisexual, Transgender/Transsexual, and Queer/Questioning (LGBTQ) Populations. CA Cancer J Clin (2015) 65:384–400. doi: 10.3322/caac.21288 26186412 PMC4609168

[9] Griggs J Maingi S Blinder V Denduluri N Khorana AA Norton L. American Society of Clinical Oncology Position Statement: Strategies for Reducing Cancer Health Disparities Among Sexual and Gender Minority Populations. J Clin Oncol (2017) 35(19):2203–8. doi: 10.1200/JCO.2016.72.0441 28368670

[10] Damaskos P Amaya B Gordon R Walters CB. Intersectionality and the LGBT Cancer Patient. Semin Oncol Nursing (2018) 34(1):30–6. doi: 10.1016/j.soncn.2017.11.004 PMC7424551 29325815

[11] Bowleg L. When Black = Lesbian = Woman = Black Lesbian Woman: The Methodological Challenges of Qualitative and Quantitative Intersectionality Research. Sex Roles (2008) 59:312–25. doi: 10.1007/s11199-008-9400-z

[12] Bryson MK Taylor ET Boschman L Hart TL Gahagan J Rail G. Awkward Choreographies From Cancer's Margins: Incommensurabilities of Biographical and Biomedical Knowledge in Sexual and/or Gender Minority Cancer Patients’ Treatment. J Med Humanit (2018) 41(3):341–61. 10.1007/s10912-018-9542-0 PMC7343748 30488328

[13] Arthur EK Kamen CS. Hidden Patients, Hidden Partners: Prostate Cancer Care for Gay and Bisexual Men. Oncol Nurs Forum (2018) 45(4):435–8. doi: 10.1188/18.ONF.435-438 PMC6419741 29947352

[14] Girgis A Lambert S Johnson C Waller A Currow D. Physical, Psychosocial, Relationship, and Economic Burden of Caring for People With Cancer: A Review. J Oncol practice (2013) 9(4):197–202. doi: 10.1200/JOP.2012.000690 PMC3710169 23942921

[15] Perz J Ussher JM Butow P Wain G. Gender Differences in Cancer Carer Psychological Distress: An Analysis of Moderators and Mediators. Eur J Cancer Care (Engl) (2011) 20(5):610–9. doi: 10.1111/j.1365-2354.2011.01257.x 21545568

[16] Brotman S Ryan B Collins S Chamberland L Cormier R Julien D. Coming Out to Care: Caregivers of Gay and Lesbian Seniors in Canada. Gerontologist (2007) 47:490–503. doi: 10.1093/geront/47.4.490 17766670

[17] Harrington RV Lackey FN Gates FM. Needs of Caregivers of Clinic and Hospice Cancer Patients. Cancer Nursing (1996) 19(2):118–25. doi: 10.1097/00002820-199604000-00006 8635165

[18] Washington KT McElroy J Albright D Oliver DP Lewis A Meadows S. Experiences of Sexual and Gender Minorities Caring for Adults With Non-AIDS-Related Chronic Illnesses. Soc Work Res (2015) 39(2):71–81. doi: 10.1093/swr/svu030

[19] Capistrant BD Lesher L Kohli N Merengwa EN Konety B Mitteldorf D. Social Support and Health-Related Quality of Life Among Gay and Bisexual Men With Prostate Cancer. Oncol Nurs Forum (2018) 45(4):439–55. doi: 10.1188/18.ONF.439-455 PMC7201404 29947351

[20] Shiu C Muraco A Fredriksen-Goldsen K. Invisible Care: Friend and Partner Care Among Older Lesbian, Gay, Bisexual, and Transgender (LGBT) Adults. J Soc Soc Work Res (2016) 7(3):527–46. doi: 10.1086/687325 PMC5373808 28367277

[21] Kamen C. Lesbian, Gay, Bisexual, and Transgender (LGBT) Survivorship. Semin Oncol Nursing (2018) 34(1):52–9. doi: 10.1016/j.soncn.2017.12.002 PMC5811352 29275016

[22] Meyer IH. Prejudice, Social Stress, and Mental Health in Lesbian, Gay, and Bisexual Populations: Conceptual Issues and Research Evidence. Psychol Bull (2003) 129(5):674–97. doi: 10.1037/0033-2909.129.5.674 PMC2072932 12956539

[23] Mongelli F Perrone D Balducci J Sacchetti A Ferrari S Mattei G. Minority Stress and Mental Health Among LGBT Populations: An Update on the Evidence. Minerva Psichiatrica (2019) 60(1):27–50. doi: 10.23736/S0391-1772.18.01995-7

[24] Morandini JS Blaszczynski A Dar-Nimrod I Ross MW. Minority Stress and Community Connectedness Among Gay, Lesbian and Bisexual Australians: A Comparison of Rural and Metropolitan Localities. Aust New Z J Public Health (2015) 39(3):260–6. doi: 10.1111/1753-6405.12364 25904119

[25] Jabson JM Donatelle RJ Bowen D. Breast Cancer Survivorship: The Role of Perceived Discrimination and Sexual Orientation. J Cancer Surviv (2010) 5(1):92–101. 21165708 10.1007/s11764-010-0161-7

[26] Boehmer U Glickman M Milton J Winter M. Health-Related Quality of Life in Breast Cancer Survivors of Different Sexual Orientations. Qual Life Res (2012) 21(2):225–36. doi: 10.1007/s11136-011-9947-y 21660650

[27] Ussher JM Power R Perz J Hawkey AJ Allison K. The Out With Cancer Study Team. LGBTQI Inclusive Cancer Care: A Discourse Analytic Study of Health Care Professional, Patient and Carer Perspectives. Front Oncol (2022) 12. 10.3389/fonc.2022.832657 PMC9127408 35619900

[28] Jabson J Kamen CS. Sexual Minority Cancer Survivors’ Satisfaction With Care. J Psychosoc Oncol (2016) 34(1-2):28–38. doi: 10.1080/07347332.2015.1118717 26577277 PMC4916952

[29] Seay J Mitteldorf D Yankie A Pirl WF Kobetz E Schlumbrecht M. Survivorship Care Needs Among LGBT Cancer Survivors. J Psychosocial Oncol (2018) 36(4):393–405. doi: 10.1080/07347332.2018.1447528 29791273

[30] Boehmer U. LGBT Populations' Barriers to Cancer Care Seminars in Oncology Nursing. (2018) 34(1):21–9. doi: 10.1016/j.soncn.2017.11.002 29338894

[31] National Coalition of Anti-Violence Programs. Lesbian, Gay, Bisexual, Transgender, Queer, and HIV-Affected Hate Violence in 2016. New York, NY: National Coalition of Anti-Violence Programs (2016).

[32] Callander D Wiggins J Rosenberg S Cornelisse VJ Duck-Chong E Holt M. The 2018 Australian Trans and Gender Diverse Sexual Health Survey: Report of Findings. Sydney, NSW: The Kirby Institute (2019).

[33] Robinson KH Townley C Ullman J Denson N Davies C Bansel P. Advancing LGBTQ+ Safety and Inclusion: Understanding the Lived Experiences and Health Needs of Sexuality and Gender Diverse People in Greater Western Sydney. (Penrith, NSW: Western Sydney University & ACON) (2020). doi: 10.26183/mr1b-sb87

[34] Hyde Z Doherty M Tilley P McCaul K Rooney R Jancey J. The First Australian National Trans Mental Health Study: Summary of Results. Perth, Australia: School of Public Health, Curtin University (2014).

[35] Mountz SE. That’s the Sound of the Police: State-Sanctioned Violence and Resistance Among LGBT Young People Previously Incarcerated in Girls’ Juvenile Justice Facilities. Affilia: J Women Soc Work (2016) 31(3):287–302. doi: 10.1177/0886109916641161

[36] Gaynor TS. Social Construction and the Criminalization of Identity: State-Sanctioned Oppression and an Unethical Administration. Public Integrity (2018) 20(4):358–69. doi: 10.1080/10999922.2017.1416881

[37] Aggleton P Davies P Hart G. AIDS. Hoboken: Taylor and Francis (1995).

[38] Drescher J. Out of DSM: Depathologizing Homosexuality. Behav Sci (Basel) (2015) 5(4):565–75. doi: 10.3390/bs5040565 PMC4695779 26690228

[39] Bull M Pinto S Wilson P. Homosexual Law Reform in Australia. In: Trends and Issues in Criminal Justice, No 29 Canberra. (Canberra Australia: Australian Institute of Criminology) (1991).

[40] Davy Z. The DSM-5 and the Politics of Diagnosing Transpeople. Arch Sexual Behavior (2015) 44(5):1165–76. doi: 10.1007/s10508-015-0573-6 26054486

[41] Kraus C. Classifying Intersex in DSM-5: Critical Reflections on Gender Dysphoria. Arch Sexual Behavior (2015) 44(5):1–1163. doi: 10.1007/s10508-015-0550-0 25944182

[42] Human Dignity Trust. Map of Countries That Criminalise LGBT People United Kingdom: Human Dignity Trust (2022). Available at: https://www.humandignitytrust.org/lgbt-the-law/map-of-criminalisation/.

[43] Bayrakdar S King A. LGBT Discrimination, Harassment and Violence in Germany, Portugal and the UK: A Quantitative Comparative Approach Vol. 1139212110392. Current sociology (2021).

[44] Flaherty I Wilkinson J. Marriage Equality in Australia: The 'No' Vote and Symbolic Violence. J Sociology (Melbourne Vic) (2020) 56(4):664–74. doi: 10.1177/1440783320969882

[45] Poulos E. Constructing the Problem of Religious Freedom: An Analysis of Australian Government Inquiries Into Religious Freedom. Religions (2019) 10(10):583. doi: 10.3390/rel10100583

[46] Levin S. Mapping the Anti-Trans Laws Sweeping America: 'A War on 100 Fronts'. Guardian (2021).

[47] Pham A Morgan AR Kerman H Albertson K Crouch JM Inwards-Breland DJ. How Are Transgender and Gender Nonconforming Youth Affected by the News? A Qualitative Study. J Adolesc Health (2020) 66(4):478–83. doi: 10.1016/j.jadohealth.2019.11.304 31964610

[48] Hughto JMW Pletta D Gordon L Cahill S Mimiaga MJ Reisner SL. Negative Transgender-Related Media Messages Are Associated With Adverse Mental Health Outcomes in a Multistate Study of Transgender Adults. LGBT Health (2020) 8(1):32–41. 33170060 10.1089/lgbt.2020.0279 PMC7826438

[49] Detrie PM Lease SH. The Relation of Social Support, Connectedness, and Collective Self-Esteem to the Psychological Well-Being of Lesbian, Gay, and Bisexual Youth. J Homosexuality (2007) 53(4):173–99. doi: 10.1080/00918360802103449 18689197

[50] Jabson JM Donatelle RJ Bowen DJ. Relationship Between Sexual Orientation and Quality of Life in Female Breast Cancer Survivors. J Women's Health (2011) 20(12):1819–24. doi: 10.1089/jwh.2011.2921 PMC3236987 21992619

[51] Kamen C Mustian K Heckler C Janelsins M Peppone L Mohile S. The Association Between Partner Support and Psychological Distress Among Prostate Cancer Survivors in a Nationwide Study. J Cancer Surviv (2015) 9(3):492–9. doi: 10.1007/s11764-015-0425-3 PMC4510042 25603949

[52] Puckett JA Woodward EN Mereish EH Pantalone DW. Parental Rejection Following Sexual Orientation Disclosure: Impact on Internalized Homophobia, Social Support, and Mental Health. LGBT Health (2015) 2(3):265–9. doi: 10.1089/lgbt.2013.0024 26788675

[53] Ussher JM Allison K Perz J Power R The Out with Cancer Study Team. LGBTQI Cancer Patients’ Quality of Life and Distress: A Comparison by Gender, Sexuality, Age and Cancer Type. Front Oncol (2022):forthcoming. 10.3389/fonc.2022.873642 PMC9530284 36203463

[54] Ussher JM Perz J Allison K Power R Hawkey A Dowsett GW. Attitudes, Knowledge and Practice Behaviours of Oncology Health Care Professionals Towards Lesbian, Gay, Bisexual, Transgender, Queer and Intersex (LGBTQI) Patients and Their Carers: A Mixed-Methods Study. Patient Educ Counseling (2021). doi: 10.1016/j.pec.2021.12.008 34998663

[55] Allison K Ussher J Perz J Power R The Out with Cancer Study Team. “Queer People are Excellent Caregivers, But We’re Stretched So Very Thin”: Psychosocial Wellbeing and Impacts of Caregiving Among LGBTQI+ Cancer Carers. Patient Educ Couns (2022). 10.1186/s12885-023-11732-2 PMC10768402 38182998

[56] Crenshaw K. Mapping the Margins: Intersectionality, Identity Politics, and Violence Against Women of Color. Stanford Law Review (1991) 43(6):1241–99. doi: 10.2307/1229039

[57] Marecek J. Invited Reflection: Intersectionality Theory and Feminist Psychology. Psychol Women Quarterly (2016) 40(2):177–81. doi: 10.1177/0361684316641090

[58] Warner LR. A Best Practices Guide to Intersectional Approaches in Psychological Research. Sex Roles (2008) 59(5-6):454–63. doi: 10.1007/s11199-008-9504-5

[59] Hawkey AJ Ussher JM. Feminist Research: Inequality, Social Change, and Intersectionality. In: Flick U, editor. The Sage Handbook of Qualitative Research Design 1. London: Sage (2022). p. 175–93.

[60] Hankivsky O Reid C Cormier R Varcoe C Clark N Benoit C. Exploring the Promises of Intersectionality for Advancing Women's Health Research. Int J Equity Health (2010) 9(1):5. doi: 10.1186/1475-9276-9-5 20181225 PMC2830995

[61] Berger R. Now I See it, Now I Don’t: Researcher’s Position and Reflexivity in Qualitative Research. Qual Res (2015) 15(2):219–34. doi: 10.1177/1468794112468475

[62] Teti M Majee W Cheak-Zamora N Maurer-Batjer A. Understanding Health Through a Different Lens: Photovoice Method. In: Liamputtong P, editor. Handbook of Research Methods in Health Social Sciences. Singapore: Springer (2019).

[63] Ussher JM Hawkey A Perz J Liamputtong P Sekar J Marjadi B. Crossing Boundaries and Fetishization: Experiences of Sexual Violence for Trans Women of Color. J Interpersonal Violence (2022) 37(5-6):NP3552–NP84. doi: 10.1177/0886260520949149 32783523

[64] Ussher JM Hawkey A Perz J Liamputtong P Sekar J Marjadi B. Gender Affirmation and Social Exclusion Amongst Trans Women of Color in Australia. Int J Transgender Health (2021) 23:79–96. 10.1080/26895269.2021.1947432 PMC8986236 35403115

[65] McIntyre A. Participatory Action Research. Qualitative Research Methods Series 52. Thousand Oaks, CA: Sage (2008).

[66] Braun V Clarke V. Reflecting on Reflexive Thematic Analysis. Qual Res Sport Exercise Health (2019) 11(4):589–97. doi: 10.1080/2159676X.2019.1628806

[67] Mouzos J Thompson S. Gay-Hate Related Homicides: An Overview of Major Findings in New South Wales. Trends and Issues in Crime and Criminal Justice (2000) No 155, P 1–6.

[68] Independent Forensic Expert G. Statement on Conversion Therapy. J Forensic Leg Med (2020) 72:101930. doi: 10.1016/j.jflm.2020.101930 32452446

[69] Carpenter M. The “Normalization” of Intersex Bodies and “Othering” of Intersex Identities in Australia. Bioethical Inquiry (2018) 15(4):487–95. doi: 10.1007/s11673-018-9855-8 29736897

[70] Greenberg JA. Health Care Issues Affecting People With an Intersex Condition or DSD: Sex or Disability Discrimination. Loyola Los Angeles Law review (2012) 45(3):849.

[71] Russell GM Bohan JS McCarroll MC Smith NG. Trauma, Recovery, and Community: Perspectives on the Long-Term Impact of Anti-LGBT Politics. Traumatology (2011) 17(2):14–23. doi: 10.1177/1534765610362799

[72] Correro AN 2nd Nielson KA. A Review of Minority Stress as a Risk Factor for Cognitive Decline in Lesbian, Gay, Bisexual, and Transgender (LGBT) Elders. J Gay Lesbian Ment Health (2020) 24(1):2–19. doi: 10.1080/19359705.2019.1644570 33014237 PMC7531820

[73] Office of the Council of Europe Commissioner for Human Rights. Human Rights of LGBTI People in Europe: Current Threats to Equal Rights, Challenges Faced by Defenders, and the Way Forward. (Strasbourg: Council of Europe) (2021). Contract No.: CommDH(2021) 32.

[74] Verrelli S White FA Harvey LJ Pulciani MR. Minority Stress, Social Support, and the Mental Health of Lesbian, Gay, and Bisexual Australians During the Australian Marriage Law Postal Survey. Aust Psychol (2020) 54(4):336–46.

[75] Frost DM Fingerhut AW. Daily Exposure to Negative Campaign Messages Decreases Same-Sex Couples’ Psychological and Relational Well-Being. Group Processes Intergroup Relations (2016) 19(4):477–92. doi: 10.1177/1368430216642028

[76] Gonzalez KA Ramirez JL Galupo MP. Increase in GLBTQ Minority Stress Following the 2016 US Presidential Election. J GLBT Family Stud (2018) 14(1-2):130–51. doi: 10.1080/1550428X.2017.1420849

[77] Winter S Diamond M Green J Karasic D Reed T Whittle S. Transgender People: Health at the Margins of Society. Lancet (2016) 388(10042):390–400. doi: 10.1016/S0140-6736(16)00683-8 27323925

[78] Kerr L Fisher CM Jones T. “I’m Not From Another Planet”: The Alienating Cancer Care Experiences of Trans and Gender-Diverse People. Cancer Nursing. (2021) 1(6):E438–46. doi: 10.1097/NCC.0000000000000857 32694280

[79] Shires DA Jaffee K. Factors Associated With Health Care Discrimination Experiences Among a National Sample of Female-To-Male Transgender Individuals. Health Soc Work (2015) 40(2):134–41. doi: 10.1093/hsw/hlv025 26027422

[80] Balik CHA Bilgin H Uluman OT Sukut O Yilmaz S Buzlu S. A Systematic Review of the Discrimination Against Sexual and Gender Minority in Health Care Settings. Int J Health Services (2020) 50(1):44–61. 10.1177/0020731419885093 31684808

[81] Robinson K H Bansel P Denson N Ovenden G. Growing Up Queer: Issues Facing Young Australians Who Are Gender Variant and Sexuality Diverse. In: Young and Well Cooperative Research Centre (2014) (Melbourne: Young and Well Cooperative Research Centre. Report No.: 9780987117984.

[82] Rose D Ussher JM Perz J. Let’s Talk About Gay Sex: Gay and Bisexual Men’s Sexual Communication With Healthcare Professionals After Prostate Cancer. Eur J Cancer Care (2017) 26:e12469. 10.1111/ecc.12469 26918877

[83] Green D Parra L Blosnich J Goldbach J. Experiences of Minority Stress and Access to Primary Care Services Among Sexual Minority Adults in the United States. J Gay Lesbian Soc Serv (2022) 1–19. doi: 10.1080/10538720.2022.2044953

[84] Zimet GD Dahlem NW Zimet SG Farley GK. The Multidimensional Scale of Perceived Social Support. J Pers Assess (1988) 52(1):30–41. doi: 10.1207/s15327752jpa5201_2 2280326

[85] Durso L Meyer I. Patterns and Predictors of Disclosure of Sexual Orientation to Healthcare Providers Among Lesbians, Gay Men, and Bisexuals. Sexuality Res Soc Policy (2013) 10(1):35–42. doi: 10.1007/s13178-012-0105-2 PMC3582401 23463442

[86] Lepore S. Social Conflict, Social Support, and Psychological Distress: Evidence of Cross-Domain Buffering Effects. JJJop Psychol s (1992) 63(5):857. doi: 10.1037/0022-3514.63.5.857 1447698

[87] Stinchcombe A Smallbone J Wilson K Kortes-Miller K. Healthcare and End-Of-Life Needs of Lesbian, Gay, Bisexual, and Transgender (LGBT) Older Adults: A Scoping Review. Geriatrics (Basel) (2017) 2(1):13. doi: 10.3390/geriatrics2010013 PMC6371094 31011023

[88] LGBT Cancer Network. LGBT Caregivers. Available at: https://cancer-network.org/cancer-information/lgbt-caregivers/.

[89] Muraco A Fredriksen-Goldsen K. "That's What Friends do": Informal Caregiving for Chronically Ill Midlife and Older Lesbian, Gay, and Bisexual Adults. J Soc Pers Relat (2011) 28(8):1073–92. 10.1177/0265407511402419 PMC4013093 24817778

[90] Taylor V Whittier N Morris AD Mueller C. Collective Identity in Social Movement Communities: Lesbian Feminist Mobilization. In: Social Perspectives in Lesbian and Gay Studies: A Reader, vol. p. . London: Routledge (1992). p. 349–65.

[91] Gossett R Stanley EA Burton J. Trap Door : Trans Cultural Production and the Politics of Visibility. Cambridge, Massachusetts: The MIT Press (2017).

[92] Tran D Sullivan CT Nicholas L. Lateral Violence and Microaggressions in the LGBTQ+ Community: A Scoping Review. J Homosex (2022) 1–15. doi: 10.1080/00918369.2021.2020543 35007475

[93] Snapp SD Watson RJ Russell ST Diaz RM Ryan C. Social Support Networks for LGBT Young Adults: Low Cost Strategies for Positive Adjustment. Family relations (2015) 64(3):420–30. doi: 10.1111/fare.12124

[94] Cover R Aggleton P Rasmussen ML Marshall D. The Myth of LGBTQ Mobilities: Framing the Lives of Gender- and Sexually Diverse Australians Between Regional and Urban Contexts. Cult Health Sex (2020) 22(3):321–35. doi: 10.1080/13691058.2019.1600029 30977702

[95] Emlet CA Jung H Kim H-J La Fazia DM Fredriksen-Goldsen K. Determinants of Physical and Mental Health Among LGBT Older Adult Caregivers. Alzheimer's Dementia (2022) 17(S7):e056620.

[96] Syrowatka A Motulsky A Kurteva S Hanley JA Dixon WG Meguerditchian AN. Predictors of Distress in Female Breast Cancer Survivors: A Systematic Review. Breast Cancer Res Treat (2017) 165(2):229–45. doi: 10.1007/s10549-017-4290-9 PMC5543195 28553684

[97] McLean LM Jones JM. A Review of Distress and its Management in Couples Facing End-of-Life Cancer. Psycho-Oncology (2007) 16(7):603–16. doi: 10.1002/pon.1196 17458836

[98] Matthews AK Hotton A Li C-C Miller K Johnson A Jones KW. An Internet-Based Study Examining the Factors Associated With the Physical and Mental Health Quality of Life of LGBT Cancer Survivors. LGBT Health (2015) 3(1):65–73. 26789396 10.1089/lgbt.2014.0075

[99] Balsam KF Molina Y Beadnell B Simoni J Walters K. Measuring Multiple Minority Stress: The LGBT People of Color Microaggression Scale. Cultural Diversity Ethnic Minority Psychol (2011) 17(2):163–74. doi: 10.1037/a0023244 PMC4059824 21604840

[100] Quinn GP Alpert AB Sutter M Schabath MB. What Oncologists Should Know About Treating Sexual and Gender Minority Patients With Cancer. JCO Oncol practice (2020) 16(6):309–16. doi: 10.1200/OP.20.00036

[101] Pratt-Chapman ML. Efficacy of LGBTQI Cultural Competency Training for Oncology Social Workers. J psychosocial Oncol (2021) 39(1):135–42. doi: 10.1080/07347332.2020.1821862 33030124

[102] Seay J Hicks A Markham MJ Schlumbrecht M Bowman-Curci M Woodard J. Web-Based LGBT Cultural Competency Training Intervention for Oncologists: Pilot Study Results. Cancer (2020) 126(1):112–20. doi: 10.1002/cncr.32491 31524952

