# Supplementary material for: “Surviving Discrimination by Pulling Together”: LGBTQI Cancer Patient and Carer Experiences of Minority Stress and Social Support
Source: Front Oncol. 2022 Jun 24;12:918016. doi: 10.3389/fonc.2022.918016 (PMC9263127; doi:10.3389/fonc.2022.918016)
Supplement: Supplementary file 1 [file Table_1.pdf]

Table S1. Participant quotes

3.1 <u>Living with LGBTQI prejudice when we were younger: An era of "open discrimination and hostility"</u>

1. I am about to turn 60, when I came out in my early 20's there was a lot of open discrimination and hostility. (Survey, 57, lesbian, carer of wife with breast cancer)

2. A lot of us LGBT, some of us a good deal of us have had traumas in our background. And, um. a lot haven't, but a good deal of us have. And so, um, I guess it's all part of our journey with our sexual identity as well. Because we've got to be whole. (Shirley, 65, lesbian, carer of friend with breast cancer)

3. I got bullied at school for being gay. (Survey, 19, lesbian, carer of father with prostate cancer)

4. I suppose the bulk of it has always been from childhood growing up in a regional country town. I had homophobia but it's really misogyny, kind of putting down anything feminine as worthless. So, I mean even at those ages I was very aware of my peers and their wanting to distance themselves from who I was and what I might have represented to them. Parents had reactions to their kids wanting to play with me. And although I was always told kids they're just kids grow out of it. It wasn't just kids. It was parents and teachers. Instances of homophobia from the age of 20 onwards have been more isolated to groups of young men, calling out names so something like that. (Andrew, 48, queer, skin cancer)

5. Always had low self-esteem. Directly related to the way l was treated when young i.e. bullied for being effeminate. (Survey, 63, gay, bladder)

6. I have been verbally abused. I've been assaulted seriously in last 24 months 6 times. (Survey, 53, trans woman, soft tissue cancer)

7. Have been gay bashed twice in 20 odd years. (Survey, 47, gay, brain)

8. I have experienced discrimination a lot in my life as being out as a lesbian - from my family to society. (Survey, 67, lesbian, breast)

9. When I came out it was the early 80s. So, it was still illegal to be gay. (Finn, 56, gay, throat)

10. It used to be we need to have gay rights. Which were human rights because in Victoria and New South Wales every state basically it was illegal to be gay it was illegal for the first seven years of my relationship with [husband] to be gay and we could have gone to jail. And so, having received legal sanction if you like to our relationship no longer defined as a mental illness was for us the greatest milestone. (Anthony, 65, gay, carer of husband with prostate cancer)

11. My parents had also consented to my having ECT [Electroconvulsive therapy] as ex-gay therapy for my body dysphoria and sinful sexual desires (attracted to women). (Survey, 54, trans man, queer, thyroid)

12. Assigned female through medical violence (Survey, 34, intersex, non-binary/gender fluid, gay, medical intervention)

13. [We've had] forced [sex] assignments. Some of us have had gonadectomies, clitorectomies, hysterectomies & mastectomies as preventative measures, but often ill-advised from the archaic attitudes from the 50's & 60's aka the John Money program which still has far reaching consequences. (Survey, 57, pansexual, non-binary, intersex, skin cancer)

14. I mean sort of people in their 50s and 60s, even if they knew gay people, they wouldn't necessarily have known that they knew them because people were much more closeted. You know it was illegal. Everything was much more hidden. (Tracy, 52, lesbian, carer of partner with breast cancer)

15. I mean, when I was first gay, it was still illegal. It was such a big deal. And you would be at threat of losing your job, your housing and definitely family. (Lucinda, 59, queer, carer of partner with ovarian cancer)

16. It was hard to come out as a gay man, there was shame and guilt around that. And then when I was HIV positive, there was like another layer of shame and guilt. And then when I got the throat cancer and realized it was HPV and related to sexual activity, I felt this funny little moment when a friend said to me "what sort of cancer is it?" and I said "HPV" and he said "Oh, that was from sucking too much cock". And he said it as a joke, and I know it was a joke and I took it as a joke. But there was this moment of, I've brought this on myself. (Finn, 56, gay, throat)

17. It was up until I was 18, up until 1967, it was actually illegal to have gay sex at any age. Then it was raised to 21 then. I was brought up- I mean, I use the term being gay was unthinkable. I don't mean the cliche unthinkable. I mean, it literally was. I'm a working class lad. I mean, not quite a respectable working class lad. You know what I mean, but it was literally unthinkable (Troy, 71, gay, prostate)

3.2 <u>The legacy of discrimination: Fear of violence and hostility in the present time</u>

18. I certainly think that our experiences when we were younger shaped some of our responses to things now, maybe not always for the best. (Ross, 35, bisexual, carer of partner with brain cancer)

19. Being more effeminate I am easily spotted as gay. This can place me at greater social risk of risk. I would say I am fearful of violence. (Survey, 69, gay, skin)

20. When you grow up as a lesbian and the age group that I grew up, your attitudes and the way you deal with the world because of the condemnation you know, you had to hide things, hide yourself or not be yourself. Or you'd be yourself but you've got to be prepared for hostility. (Bernice, 61, lesbian, breast)

21. The high rates of cancer in our community - for me, as an older lesbian, never having had a public health campaign targeting our lesbian community, I can only speak to this as a lesbian. The high risks we have are linked to internalized oppression and everyday oppression e.g. smoking, drinking, drug use, exercise etc. (Survey, 67, lesbian, breast).

22. Being part of a marginalized community brings additional pressures and stresses, and the anticipation of potential discrimination, or everyday misunderstanding, is always there. This creates additional burdens which impact on health and wellbeing. This awareness needs to be out there. (Survey, 53, lesbian, breast)

23. There's still that kind of low level, just under the skin awareness that 'do I need to be careful about who I talk to you about being gay?' [Finn, 56, gay, head/neck]

24. I'm judging all the time so that I can act appropriately to be safe. When you've feared for your life at different times because of your sexuality, you carry that with you for the rest of your life. (Claire, 66, lesbian, carer of partner with ovarian cancer)

3.3 <u>"It still goes on": Living with discrimination and prejudice today</u>

25. Learn to embrace it. Learn to. Enjoy it, but also learn to survive. And, you know, hey, man, you know that the 1990s they were throwing gay men off the cliffs in Sydney. You know, like it still goes on. You know, we face discrimination and it's only a discrimination that only gay people can fully understand. (Glenn, 66, gay, head/neck)

26. I am more than happy being me, but I am treated abominably by some of my family. Totally degrading and frankly inhumane. (Survey, 61, bisexual, intersex, carer of partner with breast cancer)

27. Yeah things are better but also it's just really ominous. I mean with the redneck thing with the religious freedoms thing that's been burbling because it's all a revenge tactic for you gays and lesbians who got your way. (Raymond, 55, gay, prostate)

28. What I find happens is I kind of going through the ups and down cycles. So if there's a lot of transphobia in the media or at the time. I can I'm really, really sensitive person and I tend to get quite distressed and upset. (Oliva, 30, bisexual, trans woman, skin cancer)

29. I know that it [marriage equality debate] was incredibly tough for so many people. We had some really awkward conversations with [partner]'s parents and I saw the stuff online and of course, the work that I do, it was always there in my face type thing. (Cameron, 38, non-binary, queer, carer of partner with breast cancer)

30. My treatment was slap bang in the marriage equality. I'll share with you about what it was like being sick in a life and death situation with that bloody marriage equality stuff happening. That was very stressful when you are sick and having to listen to that stuff. (Glenn, 66, gay, head/neck)

3.4 <u>"The possibility of being judged": Fear of exclusion and discrimination in medical care</u>

31. It exacerbates people who are tunnel visioned and hateful, because of how things have gone it sort of riles them up and they think, "Oh yeah I don't like them either. Maybe I won't do certain services." And it starts making people think they can be really nasty. They'll think, "I will show those bloody pooftas". (Raymond, 55, gay, prostate)

32. Disclosing this always comes with trepidation (Survey, 49, lesbian, endometrial)

33. Because I did not disclose my sexuality, always assumed that I was straight. I had experienced quite a lot of discrimination in my professional life as a health care professional

particularly in the 80s and 90s. Makes one wary of self-disclosure in a non-capital city. (Survey, 68, lesbian, breast)

34. Although I was worried about possible discrimination given especially at the time [when] some doctors in Australia were openly against marriage equality, I was open about who I was and my relationship. However, I did discuss with my GP who was the appropriate urologist to see. (Survey, 61, gay, prostate)

35. I had bad experiences with doctors around the time of my diagnosis so I try to avoid discussing it with medical professional except where it's clinically relevant. (Survey, 48, queer, non-binary/ gender fluid, intersex, medical intervention)

36. It can be challenging having to "come out" with every different medical practitioner about being gay, so sometimes I do not refer to it, even though it might be relevant. (Survey, 71, gay, prostate)

37. I had already explained that I'm Mx. [participant's name] because I'm non-binary, not a woman. I thought to myself in that moment, I said, "Fuck this, I'm not going to have cancer and be misgendered, enough. (Flynn, 34, queer, non-binary, uterine)

3.5 Treated abominably": Rejection and prejudice impacts on social support

38. Some people just don't like us, includes family, siblings wouldn't support me when adult son died because of being a lesbian, I choose not to interact with them now, there have been bad work experiences, couple good friends if I talk about personal feelings etc. they don't call as often. Most of lesbian friends are online, it's a lonely world. (Survey, 65, lesbian, lung cancer)

39. We [my younger brother and I] are both concerned my parents and my older brother and his wife would probably disown me and my kids. So that's, I think, a realistic concern. (Mariam, 51, bisexual, multiple)

40. My wife is not working. She has some mental health issues especially around anxiety that make it hard for her to interact with other people and to go out to find a job. And so she's mostly at home and doesn't work. So if something were to happen to me it's very stressful being the only breadwinner. It's just sometimes it's stressful if I'm if I'm not able to do what I need to do. It puts us at jeopardy because we don't have security from our parents as some people in our generation might because we're gay. (Anita, 34, lesbian, uterine)

41. I was not able to leave my partner alone for the last 8 months and I felt restless physically all of time. My partner's ability to communicate was affected and I missed our ability to talk. The carers changed most days so I felt uncomfortable leaving my partner in their care. Most of my partner's family did not assist in her care and this was emotionally distressing. (Survey, 63, lesbian, carer of partner with brain cancer)

42. Well, I don't particularly have anyone to rely on for much care or help, so it's just me dealing with it when I need to go to appointments, [or] be in hospital. I've read this is common with aging intersex ppl living alone with no hope of finding a suitable spouse. (Survey, 57, non-binary/gender fluid, intersex, skin cancer)

43. I have reached out to Queer activities in my nearest city, but it seems a superficial place. Lacking compassion and empathy. No "old fashioned" caring. Strangers looking out for their own needs perhaps. An awkward and uncomfortable place to finds friends really. (Shirley, 65, lesbian, carer of friend with breast cancer)

44. When I was a young man, I was illegal because I was gay. Over the years we fought for recognition as gay men. Politics have so swamped what was my world with any different group. I'm old now and am invisible to this new 'community'. (Survey, 70, gay, carer of partner with prostate cancer)

3.6 Resisting the margins: Social support and activism buffers the negative effects of minority stress

3.6.1 Help in the most magnificent ways: Family, friends and community support

45. I have a very large support network available to me. (Survey, 42, lesbian, trans woman, skin cancer)

46. I think I might have had an unprecedented experience, because I have received unconditional and full support from all of my family, friends, acquaintances and work colleagues. (Survey, 42, trans woman, lesbian, skin cancer)

47. Each of them seem to have their different ways which is really nourishing for someone like me. It's good to have support people who include me in different ways. (Anita, 40, non-binary, bisexual, unknown primary cancer)

48. The situation has revealed new depths of connection, love and respect between families and friends. (Survey, 58, gay, carer of partner with lung cancer)

49. So I guess when I was diagnosed, my partner sort of dealt with most of coordinating the right professionals for my care because she is a doctor. She went to all the appointments with me. She sort of paved the way a lot of the time for us going into that interaction. (Ellen, 36, lesbian, breast)

50. I've been with my partner for over 20 years. So of course we see the same GPs. We are regularly, you know, going on hospital visits and that sort of thing together. And every aspect of our lives are inextricably intertwined. I do wonder how that would look if I was single, because then you don't have that person sitting next to you, and you have to actually explain. Because it still matters even if there's a person sitting next to you or not. (Barbara, 48, lesbian, endometrial)

51. People survive longer when they've got things like cancer if they've got family support. [But] when you've crossed the line, when you're so wrong in society's mind that your family are allowed to disown you and they do it, then it's done. it's not just fighting. When you pick up the phone and say, "I'm dying", they say, "Well, serves your bloody right" and you don't get the support. (Scott, 55, gay, trans man, multiple cancers)

52. "I am grateful of my mother's love and care and steadfastness" (Ben, 60, gay, head/neck)

53. Having cancer increased my family's respect and perseverance of me (Survey, 67, gay, caring for partner with prostate cancer)

54. I have grown closer to my family, sisters, and my lesbian friends. One heterosexual friend has been extremely supportive. (Survey, 75, lesbian, carer of partner with breast cancer)

55. It was my chosen family that were the ones that were there for me. (Catherine, 61, bisexual, vulval)

56. Knowing I could do this. Realizing our lesbian community where there to help out in the most magnificent ways. (Survey, 67, lesbian, carer of friend with ocular cancer)

57. It isn't a dynamic of me being a carer and everyone else being my support as a carer - everyone in my partner's support network is someone who shares the load of caring for one another, including my partner and including me. (Survey, 27, queer, non-binary, carer of partner with cervical cancer)

58. She [dog] has been the most incredible companion for me. She never fails, never ever fails. She always waits patiently for me and she was a really good focal point for me to take my eyes off myself and all the side effects I had to deal with after the operation. Just having something to look after, like her, It's a responsibility. (Ben, 60, gay, head/neck)

59. My dog was there with me all the time. So when my partner was at work, she was always by my side. [Finn, 56, gay, throat]

60. My dogs are a constant company. I don't know why, it was so good when the dogs were around. And so, you know, when I'd come home, they're always there and check out where you're going, what you're doing. [Eva, 61, lesbian, uterine]

3.6.2 Collective action to resist LGBTQI discrimination and exclusion in cancer

61. Having this cancer has made me realize I will never be put in the closet again. (Survey, 31, pansexual, trans woman, brain)

62. I have become more politically involved arguing against the proposed employment restrictions mooted in the Religious Discrimination Bill. This is because the skills and capacity to care to save my partner's life is not dependent on sexuality. (Survey, 64, gay, carer of partner with breast cancer)

63. Because of these intersections (ovarian cancer survivor, transgender man and social worker) I am very vocal about my experience and provide presentations to colleagues with the aim to increase understanding of working with transgender cancer patients. (Survey, 47, trans man, ovarian)

64. Every time I come into contact with a new healthcare professional or the introduction of a new system etc., I have to 'come out' again. I do this for several reasons, firstly there is not a way of recording this, so I never have to tell anyone. Secondly, as someone who runs a LGBTQ cancer support group I feel it is my duty to raise the profile of LGBTQ issues and make healthcare professionals away of the differences of experience. But also my journey, treatment and outcomes could be undermined by not having this information on the table as part of my 'profile'. (Survey, 61, gay, prostate)

65. My care is good generally and is good because I am so open and 'challenge' the systems that I come in touch with. I do this for myself but more for those others who might not have a voice to speak out. (Survey, 61, gay, prostate)

66. So, it started simply because there was a lack of representation and support [groups] that were already out there. I had been to a few centers and seen no support. I'd looked at loads of stuff online, seen nothing- the only stuff I'd found was like, prostate cancer specific things, or it was like, online forums that no one really managed or checked. And I sort of had enough, so I went into a [CHARITY] center and I just said to them, I was like, "You're not gonna do it. So let me do it". (Dylan, 32, gay, non-binary, leukemia)

67. I managed to get funding, it was six or seven and a half years ago. We approached [organization names] and we got funding and the organization I work for said that they were looking for someone for the group, which I wasn't at that time thinking, oh yes, you know, this is what I wanted to do. And they said, you know, apply for it. And I applied and um, I've been the coordinator of it ever since. (Leonard, 58, gay, prostate)

68. One of the reasons why we start the [support group] is once you've been through it, and the person that I started it with, he's a gay man, he also had bowel cancer, he went through it from his male viewpoint and I went through it from my female viewpoint. Yeah just so we could actually get out there and be of help, use our experience to be of help. (Paulette, 67, lesbian, colorectal)

69. Cancer made me start a charity to support other LGBTIQ cancer patients. I am designing leaflets to give specifics about the risks of our population so that queer people realize that cancer is an issue in our community. I host a peer support service for LGBTIQ people which is non tumor or gender specific. I do these things because they weren't there for me so I want to make sure they can be there for someone else. (Survey, 35, gay, non-binary/gender fluid, leukemia)

70. We produced a booklet on gay and lesbian people with cancer, which we worked fairly hard on, which told the stories about a dozen people in the gay and lesbian community who had cancer. (Lincoln, 69, gay, prostate)
